# The Porcine Nasal Microbiota with Particular Attention to Livestock-Associated Methicillin-Resistant *Staphylococcus aureus* in Germany—A Culturomic Approach

**DOI:** 10.3390/microorganisms8040514

**Published:** 2020-04-04

**Authors:** Andreas Schlattmann, Knut von Lützau, Ursula Kaspar, Karsten Becker

**Affiliations:** 1 Institute of Medical Microbiology, University Hospital Münster, 48149 Münster, Germany; andreas.schlattmann@ukmuenster.de (A.S.); knutlvl@gmail.com (K.v.L.); Ursula.Kaspar@lzg.nrw.de (U.K.); 2 Landeszentrum Gesundheit Nordrhein-Westfalen, Fachgruppe Infektiologie und Hygiene, 44801 Bochum, Germany; 3 Friedrich Loeffler-Institute of Medical Microbiology, University Medicine Greifswald, 17475 Greifswald, Germany

**Keywords:** Staphylococcus, MRSA, microbiota, pig, Macrococcus, ESBL, Enterobacterales, Firmicutes, Proteobacteria, Actinobacteria

## Abstract

Livestock-associated methicillin-resistant *Staphylococcus aureus* (LA-MRSA) remains a serious public health threat. Porcine nasal cavities are predominant habitats of LA-MRSA. Hence, components of their microbiota might be of interest as putative antagonistically acting competitors. Here, an extensive culturomics approach has been applied including 27 healthy pigs from seven different farms; five were treated with antibiotics prior to sampling. Overall, 314 different species with standing in nomenclature and 51 isolates representing novel bacterial taxa were detected. *Staphylococcus aureus* was isolated from pigs on all seven farms sampled, comprising ten different *spa* types with t899 (*n* = 15, 29.4%) and t337 (*n* = 10, 19.6%) being most frequently isolated. Twenty-six MRSA (mostly t899) were detected on five out of the seven farms. Positive correlations between MRSA colonization and age and colonization with *Streptococcus hyovaginalis*, and a negative correlation between colonization with MRSA and *Citrobacter* spp. were found (*p* < 0.05). Of 209 non-*S. aureus* members of the *Staphylococcaceae* family, 25 isolates (12.0%) from three out of the seven farms exhibited methicillin resistance, including two *Macrococcus goetzii* isolates carrying the *mecB* gene. Among 125 *Enterobacterales*, none tested positive for extended-spectrum beta-lactamase (ESBL) and carbapenemase production. The high frequency of methicillin-resistant staphylococci supports the need for enhanced efforts within the “One Health” concept to manage the antibiotic resistance crisis in the human and veterinary medicine sector.

## 1. Introduction

Resistance to antimicrobial agents in microorganisms is on the rise and poses a global health threat. The World Health Organization (WHO) estimates that deaths due to antimicrobial resistance may increase from 700,000 to 10 million by 2050 if no strategies are implemented [1]. An important pathogen in this context is methicillin-resistant *Staphylococcus aureus* (MRSA): Almost 20,000 deaths were associated with 120,000 bloodstream infections in the USA alone in 2017 [2]. While these numbers are generally lower in Germany, a mortality rate of 6.4% has still been reported for the same year by the Robert Koch Institute [3].

In order to combat colonization and eliminate reservoirs of antibiotic-resistant bacteria, novel approaches are called for. One of these is the investigation of the microbiota that could lead to discoveries with regard to synergistic or antagonistic interactions within microbial communities of certain habitats [4,5].

Habitats of particular interest are the nasal cavities of pigs because they can harbor livestock-associated MRSA (LA-MRSA). This subgroup of MRSA can colonize both humans and pigs, and occurrence of the predominant clonal complex (CC) 398 is particularly high in regions with intensive pig farming [6,7,8]. Moreover, LA-MRSA also colonizes healthy companion animals and horses without contact to livestock, which indicates that a spread to other animals has commenced [9,10,11]. Of note, zoonotic exchange of LA-MRSA between humans and animals has significant impact on the epidemiology of MRSA and contributes to the overall burden of MRSA colonization and infection in Germany and other countries [12,13,14,15,16,17].

Previous works have focused on molecular approaches, including metagenomic studies to elucidate the composition of the porcine nasal microflora. In addition to lower sensitivity, this approach does not allow for further functional metabolic characterization of hitherto undescribed bacterial taxa. We employed extensive culturomics in order to obtain data of viable, known and unknown bacterial species living in and on pig snouts. Although this approach has the caveat of not detecting microorganisms that do not survive the transport or are not able to grow on the culture media included in our design, the establishment of a biobank containing strains of the cultivable part of the porcine nasal cavity’s microbiota offers the opportunity to validly describe novel taxa and to comprehensively characterize the generated isolates and make them available to the scientific community for future research.

## 2. Materials and Methods

Samples were processed and bacterial isolates identified as described previously [18]. In brief, swab samples of healthy pigs were collected and cultured on various media. Identification was performed using matrix-assisted laser desorption/ionization time-of-flight mass spectrometry (MALDI-TOF MS) and 16S rRNA gene sequencing. Resistance profiles for staphylococci and *Enterobacterales* were determined using VITEK^®^ 2 (bioMérieux, Marcy l’Etoile, France), and PCR was performed for resistance gene detection.

Fifty-four swab samples of 27 pigs from seven farms in the Münsterland region (northwestern Germany) using the transport swabs Transwab^®^ in Amies medium (Medical Wire & Equipment, Corsham, UK) were taken and delivered within 6 h to the Institute of Medical Microbiology of the University Hospital Münster. The sampled farms were located within German post code areas of 48xxx and participating farmers filled in a data sheet for their farms regarding total number of pigs, presence of other animals, farm type, and feed type (Table 1).

Swabs were vortexed in 0.9 % saline solution and 1:10, 1:100, and 1:1000 dilutions were prepared. Columbia agar with 5% sheep blood, chocolate agar (both Becton Dickinson, Franklin Lakes, NJ, USA), Columbia CAP agar, and MacConkey agar (both Oxoid, Wesel, Germany) were inoculated with 100 µL each of each dilution and incubated aerobically for 24 h (chocolate agar: 5% CO_2_). Anaerobic incubation of Schaedler agar, Schaedler plus kanamycin/vancomycin agar (both Becton Dickinson, Franklin Lakes, NJ, USA), chocolate agar and Columbia CAP agar occurred after inoculation with the same amount of each sample for 48 h. Single colonies were picked after the respective incubation time had elapsed.

MALDI-TOFMS was applied using the Microflex LT system, Biotyper 2.0 software, and flexControl version 3.4 (Bruker Daltonik, Bremen, Germany) for primary isolate identification, as previously described [19]. Briefly, single colonies were harvested and smeared on a ground steel target plate (Bruker Daltonik) in two replicates. Samples were covered with matrix solution. After co-crystallization, they were processed in the MALDI-TOF MS using FLEXCONTROL software (Bruker Daltonik) according to the manufacturer’s instructions. Spectra were analyzed by MALDI BIOTYPER 4.1 (Bruker Daltonik) in the m/z range of 4000–10,000 Da. Identification to the species level was assumed with scores ≥ 2.0.

Extraction of DNA from bacteria with lower scores was performed with QIAamp DNA Mini Kit (Qiagen, Hilden, Germany) according to the manufacturer’s instructions. REDTaq^®^ ReadyMix™ (Sigma-Aldrich Chemie GmbH, Munich, Germany) and primers SSU-bact-27f (5′-AGA GTT TGA TCM TGG CTC AG-3′) and 16S-5 (5′-AAG GAG GTG ATC CAG CCG CA-3′) were used for 16S rRNA PCR. Gene amplification was executed at 94 °C for 5 min, 28 cycles of 45 s at 94 °C, 60 s at 60 °C and 90 s at 72 °C, and finished with the final elongation at 72 °C for 10 min. If amplification failed, primers SSU-bact-27f and 16S-2 (5′-CCG TCA ATT CMT TTG AGT TT-3′) were used with amplification conditions of 95 °C for 5 min, 30 cycles of 60 s at 95 °C, 60 s at 60 °C and 2 min at 72 °C, and finished with the final elongation at 72 °C for 10 min. The QIAquick PCR purification kit (Qiagen, Hilden, Germany) was used according to the manufacturer’s instructions to prepare the amplicons for gene sequencing with primers SSU-bact-27f and 16S-5 or SSU-bact-27f and 16S-2. Cycle sequencing technology and subsequent analysis on an ABI 3730XL (Eurofins Genomics GmbH, Ebersberg, Germany) were employed to generate chromatograms which were assembled with DNA Sequence Assembler (Heracle BioSoft S.R.L., Pitești, Romania). Sequences were deposited in GenBank under accession numbers MT007966-MT008013, MH310740 (Isolate 1a7R-KV03an), MH365349 (Isolate 1a7I-KV08an), and MH365455 (Isolate 1a6R-CH08an).

Sequence homologies of isolates <98.7% and <95% to the phylogenetically closest type species in the NCBI and Ribosomal Database Project (RDP) databases were deemed to represent candidates for new species within the genus or candidates for new genera within the family, respectively [20,21].

*Staphylococcaceae* and *Enterobacterales* isolates were subjected to phenotypic antimicrobial susceptibility testing (AST) using the VITEK^®^ 2 system with cards AST-P632 and AST-N214, respectively (all bioMérieux). *Enterococcus* isolates were screened for resistance against vancomycin, teicoplanin, linezolid, ampicillin, and imipenem using disc diffusion (Thermo Fisher Scientific, Wesel, Germany). Clinical breakpoints of the European Committee on Antimicrobial Susceptibility Testing (EUCAST) were applied to evaluate AST results [22].

Methicillin resistance conferring genes *mecA*, *mecB*, *mecC*, and *mecD* were tested for by PCR in staphylococcal isolates that tested positive for cefoxitin resistance, as previously described [23,24,25]. Isolates that tested negative were additionally tested with GenoType MRSA (Hain Liefscience GmbH, Nehren, Germany). All *S. aureus* isolates were subjected to *spa* typing [26]. Based Upon Repeat Pattern analysis was performed using StaphType software v. 2.2.1 (Ridom GmbH, Würzburg, Germany) with parameters “exclude *spa* types that are shorter than 5 repeats” and “*spa* types are clustered if costs are less or equal than 11”.

Extended-spectrum beta-lactamase (ESBL)-production was tested using MASTDISCS^TM^ ID Extended-Spektrum-β-Laktamasen (ESβL)-Set (CPD10) D67C (MAST Diagnostica, Reinfeld, Germany), carbapenemase production was tested using imipenem and meropenem disc diffusion (both Thermo Fisher Scientific).

The analysis of the association between MRSA carriage and influencing factors was carried out using Fisher’s exact test (GraphPad Prism v.5.00, GraphPad Software, La Jolla, CA, USA). Statistical significance to reject the null hypothesis was assumed at *p* < 0.05.

Type strain sequences of isolated bacteria were obtained from the SILVA rRNA database project [27]. CLUSTALW was employed for alignment of sequences; phylogenetic inferences were drawn using Kimura 2-parameter models with the maximum-likelihood method, and MEGA software was used to generate phylogenetic trees.

Similarities and differences of the porcine nasal microflora were elucidated using non-parametric multivariate statistical analysis with PRIMER v6 [28,29].

## 3. Results

### 3.1. Culturome Composition and Novel Taxa

In total, 314 microbial species with standing in nomenclature were cultivated and identified by MALDI-TOF MS and 16S rRNA gene sequencing. Additionally, 41 and 10 isolates were recovered that showed 16S rRNA gene sequence similarities below 98.7% and 95.0%, respectively, to their closest phylogenetic relatives (Supplementary Figures S1–S11), thus representing novel species and higher taxa candidates, respectively.

Of these 365 microbial species, 147 (40.2%) belonged to the phylum *Firmicutes*, 112 (30.7%) to the phylum *Proteobacteria*, 89 (24.4%) to the phylum *Actinobacteria*, 16 (4.4%) to the phylum *Bacteroidetes*, and one (0.3%) to the phylum of *Fusobacteria*.

On the genus level, the most frequently isolated bacterial taxa were *Rothia* (54/54 samples; 100%), *Staphylococcus* (52/54 samples, 96.3%), *Corynebacterium* (49/54 samples, 90.7%), *Aerococcus* and *Streptococcus* (each 44/54 samples, 81.5%).

**Figure 1:**
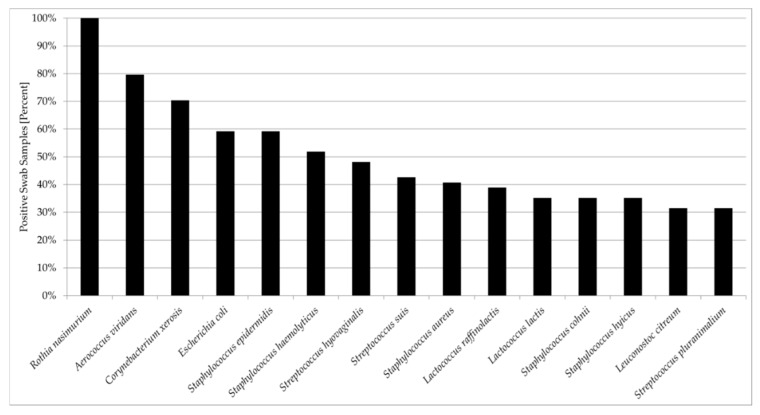
Most commonly isolated bacterial species from 54 porcine nasal swab samples.

The most frequently isolated bacterial species were *Rothia nasimurium* (54/54 samples; 100%), *Aerococcus viridans* (43/54 samples; 79.6%), *Corynebacterium xerosis* (38/54 samples; 70.4%), and *Escherichia coli* and *Staphylococcus epidermidis* (each 32/54 samples; 59.3%) (Figure 1). *Staphylococcus haemolyticus* was cultured from 28/54 (51.9%) samples, *Streptococcus hyovaginalis* from 26/54 (48.1%) samples, and *Streptococcus suis* from 23/54 (42.6%) samples. *Staphylococcus aureus* was found in 22/54 (40.7%) of swabs, *Lactococcus raffinolactis* in 21/54 (38.9%), and *Lactococcus lactis*, *Staphylococcus cohnii*, and *Staphylococcus hyicus* in 19/54 (35.2%) each. *Leuconostoc citreum* and *Streptococcus pluranimalium* were recovered from 17/54 (31.5%) samples each.

#### 3.1.1. *Firmicutes*

Of 147 bacterial taxa belonging to the phylum of *Firmicutes*, 136 (92.5%) were members of the class of *Bacilli*, four (2.7%) of the class of *Clostridia*, four (2.7%) of the class of *Tissierellia*, two (1.4%) of the class *Negativicutes*, and one (0.7%) of the class of *Erysipelotrichia*. 

Of 136 isolates found to be a member of the class *Bacilli*, 76 (56.3%) belonged to the order of *Lactobacillales* and 60 (44.1%) belonged to the order of *Bacillales*.

Of 60 bacteria belonging to the order *Bacillales*, 29 (49.2%) were members of the family *Staphylococcaceae* (for details, see below)*,* 17 (28.3%) were members of the family *Bacillaceae*, nine (15%) of the family *Planococcaceae*, four (6.7%) of the family *Paenibacillaceae*, and one (1.7%) of the family *Caryophanaceae*. Excepting *Staphylococcaceae*, Figure S1 shows the phylogenetic relationship of bacteria that were isolated from all samples belonging to these families. Isolates 6a2R-BL08, 6a1R-BL14a, and 4a1I-BL05 had less than 98.7% 16S rRNA sequence identity to their closest phylogenetic neighbors within the genera of *Bacillus*, *Caryophanon*, and *Solibacillus*, respectively.

Overall, *Staphylococcaceae* represented 7.9% of all known and unknown taxa found in this study. They comprised 22 staphylococcal and four macrococcal species. Further species belonged to the genus *Jeotgallicoccus* (*n* = 3). The phylogenetic relationship of *Staphylococcaceae* isolated from all samples is given in Figure S2. Of these, isolates 4a1I-BL30 and 3a6R-BL04 exhibited less than 98.7% 16S rRNA sequence identity to their closest phylogenetic neighbors within the genera of *Macrococcus* and *Jeotgallicoccus*, respectively.

**Figure 2:**
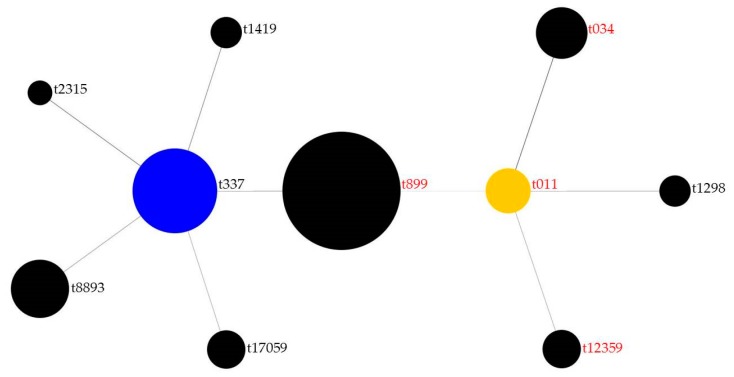
Based Upon Repeat Pattern analysis of 51 isolated *spa* types. The blue cluster “t337” is the founder of ST337, the yellow cluster “t011” is the founder of ST11; *spa* types in red text were methicillin-resistant *Staphylococcus aureus* (MRSA). Sizes of dots represent the number of isolates found in this study, connecting lines indicate evolutionary relatedness among *spa* types.

At least one pig on each farm was colonized by *S. aureus*. The *spa* types of all 51 *S. aureus* strains that were isolated from all samples were associated with clonal complex (CC) 398 and CC9; the exception is *spa* type t17059, which has not yet been subjected to multilocus sequence typing or whole genome sequencing. The detected *spa* types were t899 (*n* = 15; 29.4%), t337 (*n* = 10; 19.6%), t8893 (*n* = 6; 11.8%), t034 (*n* = 5; 9.8%), t011 (*n* = 4; 7.8%), t12359 and t17059 (each *n* = 3; 5.9%), t1298 and t1419 (each *n* = 2; 3.9%), and t2315 (*n* = 1; 2%). Figure 2 shows a Based Upon Repeat Pattern (BURP) analysis of the isolated *spa* types, indicating the long-term evolution of *S. aureus* isolates found in this study.

**Figure 3:**
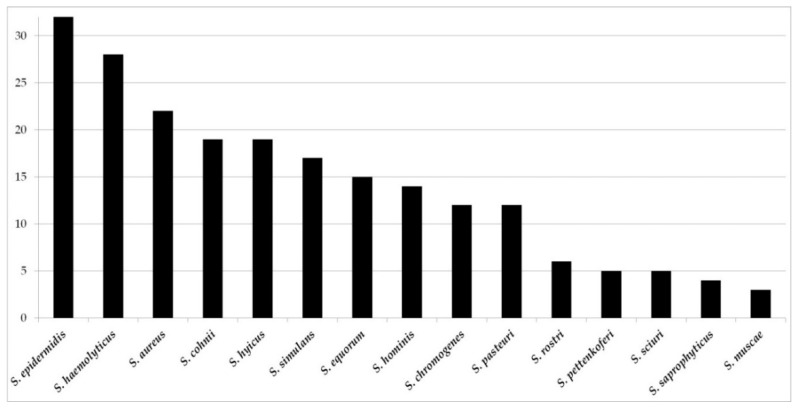
Most commonly isolated staphylococcal species from 54 porcine nasal swab samples.

On all farms, a total of 209 non-*S. aureus* members of the *Staphylococcus* genus were isolated. Most frequently, isolates of *S. epidermidis* (*n* = 35), *S. haemolyticus* (*n* = 28), *S. cohnii* (*n* = 27), *S. equorum* (*n* = 20), and *S. hyicus* (*n* = 19) were found (Figure 3). Less abundant staphylococcal isolates were *S. simulans* (*n* = 14), *S. pasteuri* (*n* = 13), *S. chromogenes* (*n* = 11), *S. sciuri* (*n* = 10), and *S. hominis* (*n* = 8). Additionally, eight other *Staphylococcaceae* were detected in the course of the investigation: four *Macrococcus* and four *Jeotgallicoccus* isolates.

Of 76 *Lactobacillales*, 13 (17.1%) were members of the family *Enterococcaceae*, 10 (13.2%) of the family *Aerococcaceae,* seven (9.2%) of the family *Leuconostocaceae*, and four (5.3%) of the family *Carnobacteriaceae*. Figure S3 shows the phylogenetic relationship of bacteria that were isolated from all samples belonging to these families. Of note, *Aerosphaera taetra* has yet to be assigned to a family within the order of *Lactobacillales*. In total, 22 isolates from five out of seven farms belonging to the genus *Enterococcus* were isolated: five *E. eurekensis*, three *E. aquimarinus* and *E. devriesei* each, two *E. faecalis*, *E. faecium*, and novel *Enterococcus* sp. each, one *E. avium*, *E. gallinarum*, *E. italicus*, *E. hirae*, and *E. casseliflavus* each.

Isolates 1a7I-CH07an, 6a6R-SA02, and 2a1R-BL19 had less than 98.7% 16S rRNA sequence identity to their closest phylogenetic neighbors within the genera of *Enterococcus*, *Aerococcus*, and *Globicatella*, respectively. Isolates 7a2I-CA08, 4a3R-BL04 (both *Facklamia*), 6a4I-CA15 (*Abiotrophia*), 2a1R-CA10 (*Globicatella*) had less than 95% 16S rRNA sequence identity to their closest phylogenetic neighbors in the family of *Aerococcaceae*. Isolates 1a7R-KV03an and 1a7I-KV08an probably lie within the order of *Tissierellales* with sequence identities below 90% to their phylogenetic closest matches with standing in nomenclature of *Tissierella creatinini* and *Sporanaerobacter acetigenes*, respectively.

Of 76 bacteria belonging to the order *Lactobacillales*, 22 (28.9%) were members of the family *Streptococcaceae* and 18 (23.7%) were members of the family *Lactobacillaceae*. Figure S4 shows the phylogenetic relationship of bacteria isolated from all samples belonging to these families. Isolates 6a4I-CA06, 2a1R-BL03, and 6a4R-CH01 had less than 98.7% 16S rRNA sequence identity to their closest phylogenetic neighbors within the genus of *Streptococcus* while isolate 4a1I-CA04 was most closely related to *Erysipelothrix inopinata* (95.55% sequence identity) and *Erysipelothrix rhusiopathiae* (95.06% sequence identity) which belong to the family *Erysipelotrichaceae*.

#### 3.1.2. *Actinobacteria*

Of 89 bacterial taxa belonging to the phylum and the class of *Actinobacteria*, 45 (50.6%) belonged to the order of *Micrococcales*, 25 (28.1%) to the order of *Corynebacteriales*, 17 (19.1%) to the order of *Actinomycetales*, and one each to the orders *Bifidobacteriales* and *Streptomycetales* (1.1% each).

All 25 isolates belonging to the class of *Corynebacteriales* belonged to the family of *Corynebacteriaceae*. Figure S5 shows their phylogenetic relationship. Isolates 1a3R-CA07, 4a1I-BL12, and 4a1R-BL13 had less than 98.7% 16S rRNA sequence identity to their closest phylogenetic relatives of *Corynebacterium vitaeruminis*, *Corynebacterium humireducens*, and *Corynebacterium pilosum*, respectively. Isolate 7a2R-anCA06 was most closely related to *Propioniciclava sinopodophylli* with 16S rRNA sequence identity below 95%. Isolate 1a6I-CH08an probably belonged to the family of *Lachnospiraceae*, with *Faecalicatena fissicatena* being its closest phylogenetic relative (93.18% 16S rRNA sequence identity). Isolate 7a1R-CH28b’s closest phylogenetic relation was *Helcococcus kunzii*.

Of the 45 taxa belonging to the order of *Micrococcales*, 22 (48.9%) were members of the family *Micrococcaceae* and 11 (24.4%) of the family *Microbacteriaceae*. Figure S6 shows the phylogenetic relationship of bacteria that were isolated from all samples belonging to these families.

Isolates 7a1I-CH13 and 6a2R-CA11 probably constituted members of the genus *Leucobacter* and isolate 4a3I-CA14 showed 98.57% sequence identity to *Microbacterium paraoxydans*. Isolate 4a1R-BL08 showed most similarities to members of the genus *Rothia* in its 16S rRNA gene sequence.

Of the 45 taxa belonging to the order of *Micrococcales*, six (13.3%) belonged to the family of *Dermabacteraceae*, four (8.9%) to the family of *Brevibacteriaceae*, and one each (2.2% each) to the families of *Sanguibacteraceae* and *Promicromonosporaceae*. Additionally, the phylogeny of 17 bacteria belonging to the order *Actinomycetales* with taxa from the families of *Propionibacteriaceae* (*n* = 9; 52.9%), *Dietziaceae* (*n* = 4; 23.6%), *Actinomycetaceae* (*n* = 3; 17.6%), and *Nocardiopsaceae* (*n* = 1; 5.9%), and one of each order of *Bifidobacteriales* and *Streptomycetales* and their respective families *Bifidobacteriaceae* and *Streptomycetaceae* are shown in Figure S7.

Isolate 4a2I-BL24 had less than 98.7% 16S rRNA sequence identity to its closest phylogenetic neighbor within the genus of *Luteococcus*. Isolate 7a1I-anCA08 was most closely related to *Isoptericola jiangsuensis* with a 16S rRNA sequence identity of 93.93%. Isolate 4a2I-BL23′s closest match was *Arcanobacterium phocae* (95.82% sequence similarity).

#### 3.1.3. *Proteobacteria* and *Bacteroidetes*

Of 112 bacterial taxa belonging to the phylum of *Proteobacteria*, 86 (76.8%) belonged to the class of *Gammaproteobacteria*, 18 (16.1%) to the class of *Betaproteobacteria*, and eight (7.1%) to the class of *Alphaproteobacteria*.

Of 86 *Gammaproteobacteria*, 41 (47.7%) belonged to the order of *Pseudomonadales* and of these, 24 (58.5%) were members of the family *Moraxellaceae* and 17 (41.5%) of the family *Pseudomonadaceae*. Figure S8 shows the phylogenetic relationship of bacteria that were isolated from all samples belonging to these families. Isolates 7a1R-BL14, 1a6I-CH20, 6a2R-MA07, 3a5I-BL02, and 6a1R-BL01 all shared most similarities in their 16S rRNA sequences with species of *Acinetobacter*, namely *A. lwoffii*, *A. piscicola*, *A. wuhouensis*, *A. junii*, and “*A. seohaensis*”, respectively. Isolate 7a2R-BL16 was probably a member of the genus *Pseudomonas*.

Of all *Gammaproteobacteria*, eight (9.3%) were members of the order *Aeromonadales* and the family *Aeromonadaceae*, four (4.7%) of the order *Xanthomonadales* and the family *Xanthomonadaceae*, three (3.5%) of the order *Pasteurellales* and the family *Pasteurellaceae*, and one (1.2%) of the order *Oceanospirillales* and the family *Oceanospirillaceae*; twenty-six (30.2%) *Gammaproteobacteria* belonged to the order of *Enterobacterales*. Of these 26, 16 were members of the family *Enterobacteriaceae*, four of the family *Yersiniaceae*, three of the family *Erwiniaceae*, and three of the family *Morganellaceae*. Three *Gammaproteobacteria* are *Wohlfahrtiimonas* spp. and have yet to be assigned to an order and a family. Figure S9 shows the phylogenetic relationship of the aforementioned bacteria that were isolated from all samples.

Isolate 1a7I-CH24 was a member of the genus *Luteimonas*. Isolate 5a1I-BL15 was most likely part of the genus *Stenotrophomonas*. Isolates 5a2R-CA12 and 6a2R-MA08 had the highest percentage identity with *Wohlfahrtiimonas populi* (97.98%) and *Wohlfahrtiimonas larvae* (97.61%), respectively, and 96.20% sequence identity to each other.

Of 18 *Betaproteobacteria*, eight (44.4%) belonged to the order *Neisseriales* and the family *Neisseriaceae*, and 10 (55.6%) to the order *Burkholderiales*. Of these 10 *Burkholderiales*, seven (70%) were members of the family *Comamonadaceae* and three (30%) of the family *Alcaligenaceae*. Of eight *Alphaproteobacteria*, three (37.5%) belonged to the order of *Caulobacterales* and the family of *Caulobacteraceae*, and four (50%) belonged to the order of *Rhizobiales*. Of these four, three (75%) were members of the family *Brucellaceae* and one (25%) of the family *Rhizobiaceae*. *Enhydrobacter aerosaccus* belongs to the order of *Rhodospirillales* (25% of *Alphaproteobacteria*) but has yet to be assigned to a family. Figure S10 shows the phylogenetic relationship of the aforementioned bacteria that were isolated from all samples.

Isolate 6a2I-BL10 phylogenetically closest match was *Comamonas thiooxydans* (98.39% sequence identity), isolate 1a6R-MA01 was most closely related to *Lampropedia puyangensis* (97.49% sequence identity). Isolates 6a6R-CH12 and 1a2R-BL09b showed the highest 16S rRNA sequence similarity to *Neisseria shayeganii* (96.83%) and *Neisseria dentiae* (97.81%), respectively.

Of 16 bacterial taxa belonging to the phylum of *Bacteroidetes*, 11 (68.8%) belonged to the class, order and family of *Flavobacteriia*, *Flavobacteriales* and *Flavobacteriaceae,* respectively; three (18.7%) to the class and order of *Bacteroidia* and *Bacteroidales*, respectively, and two (12.5%) to the class, order and family of *Sphingobacteriia*, *Sphingobacteriales* and *Sphingobacteriaceae*, respectively. Of the three taxa that belonged to the order of *Bacteroidales*, one (33.3%) belonged to the family of *Rikenellaceae*, one (33.3%) to the family of *Porphyromonadaceae*, and one (33.3%) has yet to be assigned to a family. Figure S11 shows the phylogenetic relationship of the aforementioned bacteria that were isolated from all samples. Isolate 7a1R-anCH19 may be part of a family within the order of *Bacteroidales*: Its closest phylogenetic match was *Dysgonomonas mossi* with 88.67%. Isolate 7a2I-BL11′s closest relative on basis of 16S rRNA sequence identity was *Myroides injenensis* (95.91%) while isolate 4a1I-CH08 probably belonged to the family of *Flavobacteriaceae*, with *Flavobacterium cloacae* showing the highest sequence similarity (93.66%). Isolate 1a4I-BL06a and 4a3I-BL07 were most closely related to species of the genus *Chryseobacterium*, namely *Chryseobacterium haifense* (97.66%) and *Chryseobacterium chaponense* (96.01%). Isolate 1a6R-CH08an belonged to the genus of *Anaerocella*.

### 3.2. Comparisons and Correlations of the Porcine Nasal Microbiota

**Figure 4:**
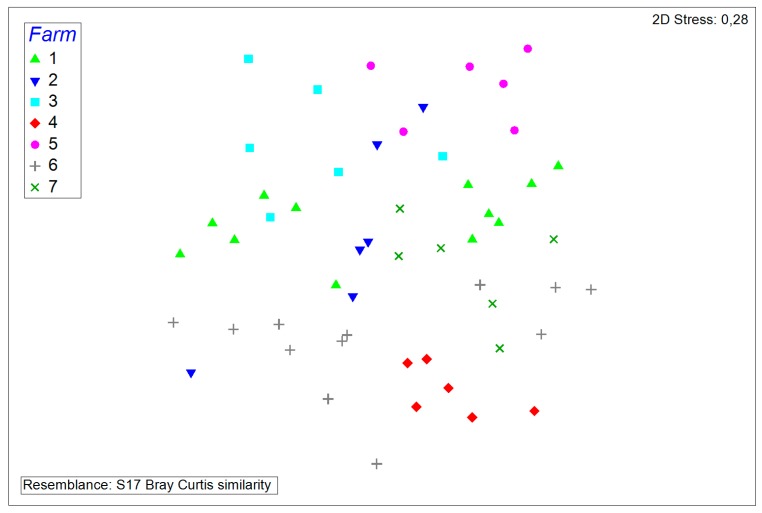
Non-metric multidimensional scaling plot of all samples. Samples from the same farms are represented by the same symbols.

The similarities of microbiota compositions were determined by non-metric multidimensional scaling plots. Samples from the same farm and of the same sampling date as is indicated by the two discernable clusters from farms #1 and #6, appear to form clusters, indicating more similarities to each other than to other samples. Samples from farm #2, however, appear to show more variations in the microflora than other samples (Figure 4).

The porcine nasal microflora is highly diverse: 183 (50.1%) of 365 bacterial taxa were isolated only once from all samples. Between 13 and 50 taxa were identified per sample, with the lowest mean of 16 from samples of farm #3 to the highest mean of 38 from samples of farm #4.

**Figure 5:**
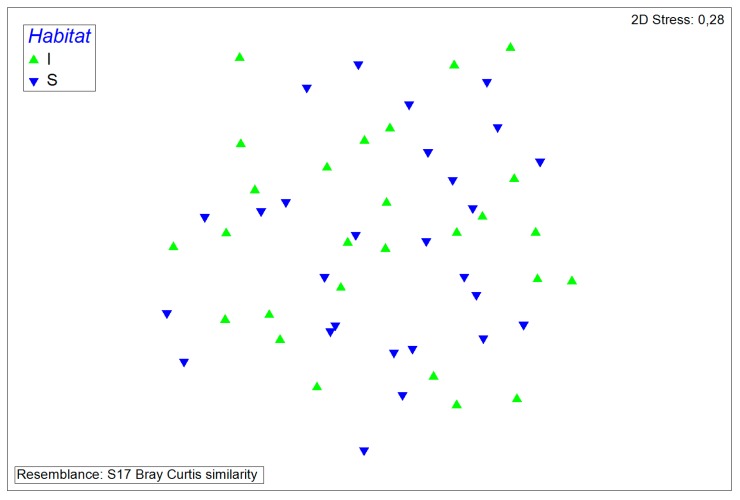
Non-metric multidimensional scaling plot of all samples. Samples from the same habitat are represented by the same symbols. “I” denotes intranasal swab samples, “S” denotes snout surface samples.

The specific habitat analyzed, i.e., the nasal cavity and surface of the pig snout, respectively, did not seem to play a major role in the composition of the porcine nasal microflora as no distinct clustering is observable in Figure 5. The mean of bacterial taxa isolated from intranasal samples was 24, the mean of snout surface samples was 26. The lowest number of taxa isolated from intranasal and snout surface swabs was 13 and 14, respectively; the highest amount was 50 and 46, respectively. This indicates similar variability among habitats.

**Figure 6:**
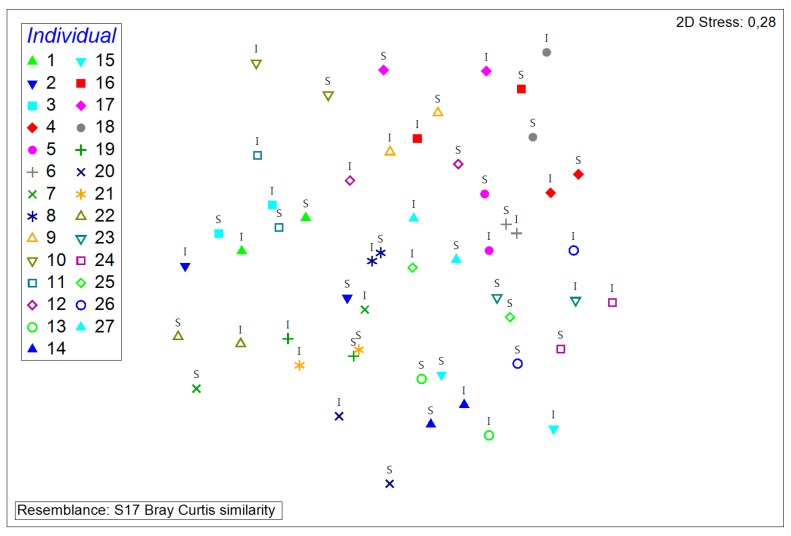
Non-metric multidimensional scaling plot of all samples. Samples from the same individual are represented by the same symbols. “I” denotes intranasal swab samples, “S” denotes snout surface samples.

A prominent factor for similarities in samples appears to be the individual itself, i.e., the swab samples from the nasal cavity and the snout surface of the same individual are on the whole relatively similar (Figure 6). However, there are samples of the same individual that are more similar to samples from two different individuals.

**Figure 7:**
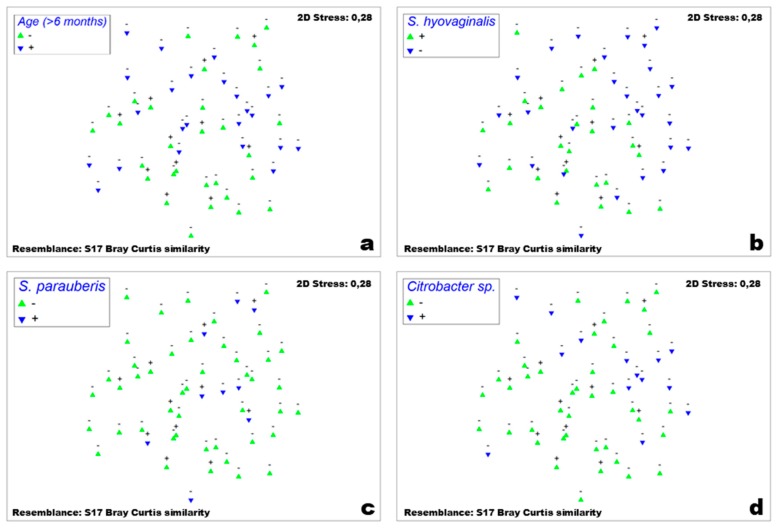
Non-metric multidimensional scaling plot of all samples. MRSA-positive samples are marked with “+”, MRSA-negative samples are marked with “−” above the symbols. (**a**) Green triangles represent samples of pigs that were six months of age or younger, blue inverted triangles represent samples of pigs that were older than six months on sampling date. (**b**) Green triangles represent samples of pigs that were positive for *S. hyovaginalis* colonization, blue inverted triangles represent samples of pigs that were negative for *S. hyovaginalis* colonization on sampling date. (**c**) Green triangles represent samples of pigs that were negative for *S. parauberis* colonization, blue inverted triangles represent samples of pigs that were positive for *S. parauberis* colonization on sampling date. (**d**) Green triangles represent samples of pigs that were positive for *Citrobacter* sp. colonization, blue inverted triangles represent samples of pigs that were negative for *Citrobacter* sp. colonization on sampling date.

Significant positively correlated co-colonization patterns of MRSA vs. age, MRSA vs. *S. hyovaginalis* (both *p* < 0.01), and MRSA vs. *Streptococcus parauberis* (*p* < 0.05) by Fisher’s exact test (Figure 7a–c). Colonization with *Citrobacter* sp. (*Citrobacter braakii*, *Citrobacter freundii*, *Citrobacter gillenii*) was statistically significant in its negative correlation with MRSA carriage (*p* < 0.05; Figure 7d). *Lactococcus lactis* narrowly failed to reach statistical significance for a negative correlation (*p* = 0.074). No correlations between the farm factors total number of pigs, presence of other animals, farm type, and feed type were found.

### 3.3. Analysis of Resistance

*S. aureus* isolates exhibiting resistance towards methicillin (*n* = 26; 51%) were detected on five out of seven farms (85.7%), all of which tested positive for *mecA* (Table 2). MRSA *spa* types were t011, t034, t899, and t12359 (Figure 2); one isolate with *spa* type t899 did not exhibit methicillin resistance or test positive for *mecA*.

Other resistances included 26 (51%) tetracycline-resistant isolates on four out of seven farms (57.1%), 10 (19.6%) clindamycin-resistant isolates on four out of seven farms (57.1%), seven (13.7%) trimethoprim/sulfamethoxazole-resistant isolates on four out of seven farms (57.1%), eight (15.7%) erythromycin-resistant isolates on three out of seven farms (42.8%), and 19 (37.2%) levofloxacin-resistant isolates from two out of seven farms (28.6%).

Phenotypic methicillin resistance with detectable *mec* resistance gene was detected in 23 *Staphylococcus* isolates (11.0%; all *mecA*) other than *S. aureus* on three out of seven farms (42.9%) (Table 3). Isolates resistant to benzylpenicillin (*n* = 66; 31.6%), clindamycin (*n* = 78; 37.3%), erythromycin (*n* = 68; 32.5%), fosfomycin (*n* = 90; 43.1%), fusidic acid (*n* = 51; 24.4%), and tetracycline (*n* = 101; 48.3%) were found on seven out of seven farms, while trimethoprim/sulfamethoxazole resistance was prevalent in seven isolates from four out of seven farms. Two *Macrococcus goetzii* isolates exhibited methicillin resistance and tested positive for *mecB*.

Screening for resistance to vancomycin, teicoplanin, linezolid, ampicillin, and imipenem in 22 *Enterococcus* isolates yielded one vancomycin-resistant *E. casseliflavus*, two ampicillin- and imipenem-resistant *E. faecium*, and one ampicillin-resistant *E. avium*.

None of the 106 *E. coli* isolates from 7/7 farms and none of the 19 *Klebsiella* isolates from five out of seven farms tested positive for either ESBL production or carbapenemase production.

Resistance to ampicillin was detected in 48/106 (45.3%) *E. coli* isolates on six out of seven farms (85.7%) (Tables S1 and S2). Of these 48 isolates, 42 (87.5%) were isolates from farms #1 (*n* = 10; 20.8%), #3 (*n* = 17; 35.4%), and #5 (*n* = 15; 31.3%), while farm #2 harbored four (8.3%) isolates and farms #4 and #6 harbored one (2.1%) isolate each. Farm #7 tested negative for the presence of ampicillin-resistant *E. coli*.

## 4. Discussion

In this study, we used a qualitative, extensive culturomics approach to examine the microbial porcine nasal flora. This in-depth approach resulted in the isolation of 51 hitherto undescribed bacterial taxa according to commonly used 16S rRNA similarity thresholds [20]. This is in contrast to previous publications in which quantitative, molecular methods were employed [30,31,32].

Lowe and colleagues defined the core microbiota in the tonsils as being dominated by *Pasteurellaceae* which were found in all twelve animals examined [30]. We have found members of this family but in far lower quantity, with eight of the 27 individuals (nine out of 54 samples) carrying *Pasteurella aerogenes*, *P. multocida*, or *Haemophilus parasuis*. Although a statistically significant correlation (*p* < 0.05) is reached for *Pasteurellaceae* carriage and age (≤24 weeks and >24 weeks), our data indicate that bacteria of this family are less likely to be found in older pigs (11 non-carriers vs. one carrier) than younger pigs (seven non-carriers vs. eight carriers). Additionally, the phylum of *Proteobacteria* and the genus *Streptococcus* mark similarities between our findings as these were isolated from all and almost all (26/27) of the sampled individuals in our study and in all of the tonsils tested by Lowe and colleagues. However, the genus *Actinobacillus,* which was highly abundant in most samples in the pyrosequencing approach, could not be isolated at all from our samples. Further discrepancies include detection of the genera *Alkanindiges*, *Peptostreptococcus*, *Veillonella*, and *Fusobacterium* which Lowe et al. detected in all samples, but we did not find at all (*Alkanindiges*, *Peptostreptococcus*) or rarely (*Veillonella*: two out of 27 individuals, two out of 54 samples; *Fusobacterium*: one out of 27 individuals, two out of 54 samples). In the same study, *Neisseriaceae* were detected in all samples; we found bacteria belonging to this genus in 13/27 animals (16/54 samples). These differences might stem from multiple reasons, be it sampling site, method used or antibiotic treatment. In the study of Lowe, tylosin, a macrolide approved only for veterinarian medicine, was administered to four out of 12 pigs in-feed, another four out of 12 pigs received additional tilmicosin, another veterinary macrolide [30]. The influence of the administered macrolides, which inhibit the growth of a broad range of Gram-positive bacteria and show less activity against Gram-negatives, may partly explain the discrepancies we found.

Weese and colleagues conducted a study in which 13/20 sampled pigs also received tylosin, but they evaluated the nasal microbiota rather than tonsillar microbiota [31]. While the dominant phylum in their data set was *Proteobacteria* as well, they reported different predominant genera: *Moraxella*, *Psychrobacter*, and *Pseudomonas* make up 71.4% of the relative abundance. We isolated bacteria belonging to these genera from 23/27 individuals (37/54 samples), indicating a high prevalence detectable by culturomics as well. However, the genera of *Acinetobacter* and *Aerococcus* which accounted for 4.8% and 1.8% of the relative abundance in the aforementioned publication, respectively, were isolated from 18/27 individuals (25/54 samples) and 26/27 individuals (44/54 samples), respectively. Despite varying overall results, the observation that two operational taxon units (OTUs) of species of lactic-acid bacteria indicate MRSA-negative pigs is to some degree similar to our finding that *Lactococcus* colonization trends toward a negative correlation with MRSA isolation. The conclusion of the authors that *Staphylococcus* was found to be an indicator OTU for MRSA-negative pigs cannot be confirmed by our results because we have found staphylococci in all individuals and almost all samples (52/54) [31]. Considering that coagulase-negative staphylococci (CoNS) from human and animal sources may harbor *mec* genes as part of mobile SCC*mec* elements and therefore may act as a reservoir for methicillin resistance, experimental approaches to conquer LA-MRSA in pigs by competitive colonization with CoNS are questionable [9,33,34,35].

The most recent study investigating the porcine nose microbiome by Strube et al. showed some differences compared to the two aforementioned studies and most similarities to our findings: *Rothia*, *Streptococcus*, *Aerococcus*, and *Staphylococcus* were found in all samples of the nasal cavity with varying abundance [32]. However, the staphylococcal species they found are differently distributed. *Staphylococcus equorum* dominated the genus of *Staphylococcus* in all samples analyzed using the *tuf*-gene approach, while we isolated this species from 11/27 individuals (15/54 samples). On the other hand, the most frequently isolated staphylococci in our culturomics approach were *S. epidermidis* (24/27 individuals; 32/54 samples) and *S. haemolyticus* (21/27 individuals; 28/54 samples). Of note, Strube and colleagues concede that the *Staphylococcus* specific primers positively biased *S. equorum* and very negatively biased *S. epidermidis* and *S. haemolyticus*. One of their results that is contrary to our data is that *Streptococcus* is “inversely associated with *Staphylococcus* and MRSA” [32]; we have shown a positive correlation between isolation of MRSA and *Streptococcus hyovaginalis* and *Streptococcus parauberis*. Furthermore, samples from which three or more streptococcal species were isolated were more likely to be positive for *S. aureus* and MRSA colonization. This may be due to entirely opposite biases in both techniques and it stresses that a combination of culture-based and molecular approaches is needed to study the microbiota composition and the interactions within as already shown for respective analyses of human microbiota [36]. It is also possible that there are species within the large and diverse genus of *Streptococcus* that either exhibit synergistic or antagonistic effects on *S. aureus* colonization. Both *S. hyovaginalis* and *S. parauberis* are associated with pigs and cows, respectively, and can therefore probably be found in samples from farms. Colonization with *Citrobacter* spp. is also common, albeit as carriers and reservoirs for resistance genes; intervention strategies employing species of this genus should be considered very carefully as this may only cause the substitution of one threat with another. Why and how the aforementioned bacterial taxa can possibly affect MRSA colonization need to be elucidated in further studies. Aside from microbial colonization, we have found a correlation between pigs older than six months and MRSA carriage. This result was indirectly supported by a study that found farm workers six times more likely to be colonized with MRSA if they worked with farrowing, i.e., older sows [37]. Whether this observation is e.g., merely the outcome of longer exposure to MRSA and therefore higher cumulative risk of carriage remains unknown; future research may shed light on this phenomenon.

All in all, differences in results are most likely due to methodological biases, antibiotic exposure, and geographic location: The studies by Lowe, Weese, and Strube were conducted in the United States, Canada, and Denmark, respectively. A potential role of bacteriocins should be further investigated as indications are given here and in the publications cited above that certain lactic-acid bacteria are inversely correlated with *S. aureus* and MRSA colonization [38]. Three years ago, a compound produced by the human nose colonizer *S. lugdunensis* called lugdunin has been isolated and shown to inhibit growth of *S. aureus* [39]. There is the possibility of microorganisms producing antimicrobials in the porcine nasal cavity as well due to relatively high colonization pressure. Considering the amount of potentially new bacterial taxa with largely unknown metabolic properties isolated by our approach, similar defense mechanisms are possible. The strain collection we have obtained from our samples will be useful in examining these hypotheses.

Seven of 27 sampled pigs (25.9%) tested positive for MRSA colonization. This is in agreement with previous studies [40]. Different *spa* types on the same farm were detected on four out of seven farms, different *spa* types from the same sample, i.e., habitats were detected in four out of 20 samples positive for *S. aureus*. Oxacillin resistance is also not evenly distributed among isolates from the same farm and in two cases not even from the same habitat. The co-colonization of isolates with different *spa* types and resistances towards oxacillin, i.e., methicillin-susceptible *S. aureus* and MRSA, has previously been described by Fetsch and colleagues; our data support their findings [41].

All levofloxacin-resistant *S. aureus* isolates were MRSA strains with the exception of those from farm #3. Additionally, the only CoNS isolate exhibiting levofloxacin resistance was an *S. hyicus* from the same farm. This is probably in direct correlation with the enrofloxacin treatment received by two of the three sampled pigs at some time point in the previous three months. Regarding resistance patterns of CoNS, we found worrying data: Schoenfelder and colleagues reported varying antibiotic resistance detection in dust and manure samples among 41 pig farms. Tetracycline was the only class to which resistance had been detected on all farms [42]; we have found CoNS resistant to tetracycline, benzylpenicillin (beta-lactam), clindamycin (lincosamide), erythromycin (macrolide), fosfomycin, and fusidic acid on all farms tested. Furthermore, we isolated at least two different CoNS species resistant towards three or more different antibiotic classes from all farms, i.e., farm #1 harbored a *Staphylococcus rostri* isolate resistant towards clindamycin, fosfomycin, and tetracycline, as well as a *Staphylococcus xylosus* isolate resistant to the same three antibiotics and fusidic acid. Nine of eleven *Staphylococcus sciuri* isolates from four out of seven farms exhibited daptomycin resistance using Vitek. Etests further indicated lowered susceptibility in seven out of nine isolates. This is alarming because the cyclic lipopeptide daptomycin is a key drug for the treatment of severe *S. aureus* infections and diminished susceptibility in CoNS has been reported on pig farms before [42]. Discrepancies in results may again stem from the type of sample used. Schoenfelder and colleagues collected environmental samples from dust and manure. It is possible that this difference accounts for the incongruous findings.

We found no evidence of ESBL-producing or carbapenem-resistant *Enterobacteriaceae*; ampicillin resistance was detected on six out of seven farms, although with vast differences in the number of isolates. One explanation for this finding is the habitat and sampling approach: studies investigating ESBL-E or carbapenemase-producing *Enterobacteriaceae* examined fecal or environmental samples that were pooled, increasing the probability to detect the bacteria in question [43]. Of note, we found the vast majority (20/21 isolates) of fluoroquinolone-resistant *E. coli* in samples from two individuals that were treated with enrofloxacin within three months for five days prior to sampling. Similarly, all *E. coli* isolates from farm #5 show resistance towards beta-lactam agents (15/48). Although less pronounced in this case because ampicillin resistance is frequently found on other farms, this was the only farm where not one isolate was found to be susceptible to a beta-lactam agent. Many factors confer quinolone resistance, at times causing resistance towards other antimicrobial agents as well [44]. These findings further underpin the vast body of evidence highlighting the importance of antibiotic stewardship in veterinary medicine that is at least in Germany starting to take effect [45,46].

## 5. Conclusions

Our approach sought to reconstruct the cultivable constituents of the porcine nasal microflora as closely as possible to natural colonization patterns. The study at hand has found *Rothia nasimurium* to be a main constituent of the porcine nasal microflora. Multiple bacterial isolates appear to constitute hitherto undescribed taxa. This fact can pose an untapped potential or unknown threat because roles as both, producer of unknown antimicrobial agents or resistance gene reservoirs are possible. The frequency of MRSA on farms is comparable to previous reports. Antibiotic resistance gene carriage by CoNS and macrococci must not be ignored and should be monitored. No ESBL-producing *E. coli* or *Klebsiella* isolates were found, possibly due to the examined habitat. It is of paramount importance to combat this threat to animal and human health care, and therefore to implement “One Health” approaches.

## Figures and Tables

**Table 1:** Data of sampled farms.

Farm	Total Number of Pigs	Additional Animals on Farm	Feed Consistency	Individual	Age in Months	Antibiotics
#1	1400	Cattle	Solid	#1	0.5	None
				#2	0.5	None
				#3	0.5	None
				#4	65	None
				#5	39	None
				#6	52	None
#2	2000	Dogs, cats	Liquid and pellets	#1	26	None
				#2	19.5	None
				#3	26	None
#3	3800	Dogs	Liquid	#1	52	None
				#2	39	Enrofloxacin
				#3	51.5	Enrofloxacin
#4	1000	Dogs, cats, horses	Liquid	#1	3	None
				#2	3	None
				#3	3	None
#5	2200	Dogs, horses	Liquid and solid	#1	2	Amoxicillin
				#2	2	Amoxicillin
				#3	2	Amoxicillin
#6	1600	Dogs	Liquid	#1	4	None
				#2	4	None
				#3	4	None
				#4	35.5	None
				#5	9.5	None
				#6	29.5	None
#7	1600	Dogs, sheep	Liquid	#1	6	None
				#2	6	None
				#3	6	None

**Table 2:** *S. aureus* isolates with *spa* types, detected *mecA* genes, and phenotypic antimicrobial susceptibility test results.

Farm	Individual	Sampling Site	*spa* Type	Resistance Gene *mecA*	Phenotypic Antimicrobial Susceptibility Test Profile ^a^		# of Isolates
					CXI Screening	Other Resistances	
#1	#1	Nasal cavity	t011	+	+	OXA, BEN, TET	2
		Snout surface	t011	+	+	OXA, BEN, TET	1
	#2	Nasal cavity	t1298	n/a	−	BEN, TET	2
			t337	n/a	−	BEN, TET	1
		Snout surface	t011	+	+	OXA, BEN, TET	1
			t337	n/a	−	BEN, TET	2
	#3	Nasal cavity	t337	n/a	−	BEN, TET	1
		Snout surface	t8893	n/a	−	BEN, TET	1
			t337	n/a	−	BEN, TET	4
			t1419	n/a	−	BEN, TET, TRS	2
#2	#2	Nasal cavity	t337	n/a	−	BEN	2
			t8893	n/a	−	BEN	2
		Snout surface	t8893	n/a	−	BEN	2
#3	#1	Snout surface	t17059	n/a	−	LEV, BEN	1
	#2	Snout surface	t17059	n/a	−	LEV, BEN	2
#4	#1	Snout surface	t8893	n/a	−	BEN	1
	#2	Snout surface	t034	+	+	CLI, ERY, BEN, TRS, TET	1
#5	#1	Nasal cavity	t034	+	+	CLI, ERY, OXA, BEN, TRS, TET	2
			t034	+	+	CLI, ERY, OXA, BEN, TET	1
		Snout surface	t034	+	+	CLI, ERY, OXA, BEN, TRS, TET	1
#6	#2	Nasal cavity	t899	+	+	CLI, LEV, OXA, BEN	1
	#3	Nasal cavity	t899	n/a	−	LEV, BEN	1
			t899	+	+	LEV, OXA, BEN	3
			t899	+	+	CLI, LEV, OXA, BEN	1
		Snout surface	t899	+	+	LEV, OXA, BEN	9
	#4	Snout surface	t2315	n/a	−	BEN	1
#7	#1	Nasal cavity	t12359	+	+	CLI, ERY, OXA, BEN, TRS, TET	1
		Snout surface	t12359	+	+	CLI, ERY, OXA, BEN, TET	2

+, positive; −, negative; n/a, not applicable. ^a^ as determined by Vitek 2 using cards AST-P632; BEN, benzylpenicillin; CLI, clindamycin; CXI, cefoxitin; ERY, erythromycin; LEV, levofloxacin; OXA, oxacillin; TET, tetracycline; TRS, trimethoprim/sulfamethoxazole.

**Table 3:** Oxacillin-resistant non-*S. aureus* staphylococci including two *Macrococcus goetzii* isolates with detected *mecA* genes and phenotypic antimicrobial susceptibility test results.

Farm	Individual	Sampling Site	Species	Resistance Gene	Resistances ^a^
#4	#1	Nasal cavity	*S. pasteuri*	*mecA*	FOS, OXA, BEN, TET
			*S. haemolyticus*	*mecA*	CLI, FOS, GEN, CXI, BEN, TET
			*S. pasteuri*	*mecA*	FOS, OXA, BEN, TET
		Snout surface	*S. hominis*	*mecA*	FOS, OXA, BEN, TET
			*S. pasteuri*	*mecA*	CLI, FOS, OXA, CXI, BEN
			*S. pasteuri*	*mecA*	CLI, FOS, OXA, CXI, BEN
	#2	Nasal cavity	*S. saprophyticus*	*mecA*	FOS, OXA, CXI, BEN, TET
			*S. pasteuri*	*mecA*	FOS, OXA, BEN, TET
			*S. epidermidis*	*mecA*	CLI, OXA, CXI, TET
		Snout surface	*S. haemolyticus*	*mecA*	CLI, FOS, GEN, CXI, BEN, TET
			*S. cohnii*	*mecA*	CLI, ERY, FUS, OXA, CXI, BEN, TRS, TET
	#3	Nasal cavity	*S. pasteuri*	*mecA*	FOS, OXA, CXI, BEN, TET
#5	#1	Nasal cavity	*S. sciuri*	*mecA*	CLI, DAP, FUS, OXA, CXI
			*S. sciuri*	*mecA*	CLI, DAP, FUS, OXA, BEN, TET
			*M. goetzii*	*mecB*	CLI, ERY, FOS, OXA, TET
		Snout surface	*M. goetzii*	*mecB*	CLI, ERY, FOS, OXA, CXI, TET
			*S. epidermidis*	*mecA*	CLI, ERY, FOS, OXA, CXI, BEN, TET
			*S. equorum*	*mecA*	CLI, ERY, FOS, OXA, CXI, BEN, TET
	#2	Snout surface	*S. epidermidis*	*mecA*	CLI, ERY, FOS, OXA, CXI, BEN, TET
			*S. sciuri*	*mecA*	CLI, DAP, ERY, FUS, OXA, CXI, BEN, TET
			*S. sciuri*	*mecA*	CLI, DAP, ERY, FUS, OXA, CXI, BEN, TET
			*S. sciuri*	*mecA*	CLI, DAP, ERY, FUS, OXA, CXI, BEN, TET
			*S. haemolyticus*	*mecA*	CLI, ERY, FOS, GEN, OXA, CXI, BEN, TET
#7	#2	Snout surface	*S. pasteuri*	*mecA*	CLI, ERY, FOS, OXA, CXI, BEN, TET
	#3	Snout surface	*S. pasteuri*	*mecA*	CLI, ERY, FOS, OXA, BEN, TET

^a^ as determined by Vitek 2 using cards AST-P632; BEN, benzylpenicillin; CLI, clindamycin; CXI, cefoxitin; ERY, erythromycin; FOS, fosfomycin; FUS, fusidic acid; LEV, levofloxacin; OXA, oxacillin; TET, tetracycline; TRS, trimethoprim/sulfamethoxazole.

## Supplementary Materials

The following are available online at https://www.mdpi.com/2076-2607/8/4/514/s1, Figure S1: Position of isolates 4a1I-BL05, 6a1R-BL14a, and 6a2R-BL08 in phylogenetic relation to closely related bacterial species also isolated from porcine nasal swab samples, Figure S2: Position of isolates 4a1I-BL30 and 3a6R-BL04 in phylogenetic relation to closely related bacterial species also isolated from porcine nasal swab samples, Figure S3: Position of isolates 1a7I-CH07an, 6a4I-CA15, 7a2I-CA08, 4a3R-BL04, 2a1R-CA10, 2a1R-BL19, 6a6R-SA02, 4a1I-CA04, 1a6I-CH08an, 1a7R-KV03an, 1a7I-KV08an, and 7a1R-CH28b in phylogenetic relation to closely related bacterial species also isolated from porcine nasal swab samples, Figure S4: Position of isolates 2a1R-BL03, 6a4I-CA06, and 6a4R-CH01 in phylogenetic relation to closely related bacterial species also isolated from porcine nasal swab samples, Figure S5: Position of isolates 4a1I-BL12, 1a3R-CA07, and 4a1R-BL13 in phylogenetic relation to closely related bacterial species also isolated from porcine nasal swab samples, Figure S6: Position of isolates 4a1R-BL08, 7a1I-CH13, 6a2R-CA11, and 4a3I-CA14, in phylogenetic relation to closely related bacterial species also isolated from porcine nasal swab samples, Figure S7: Position of isolates 7a1I-anCA08, 4a2I-BL23, 7a2R-anCA06, and 4a2I-BL24 in phylogenetic relation to closely related bacterial species also isolated from porcine nasal swab samples, Figure S8: Position of isolates 7a1R-BL14, 1a6I-CH20, 6a2R-MA07, 6a1R-BL01a, 3a5I-BL02, and 7a2R-BL16 in phylogenetic relation to closely related bacterial species also isolated from porcine nasal swab samples, Figure S9: Position of isolates 5a2R-CA12, 6a2R-MA08, 1a7I-CH24, and 5a1I-BL15 in phylogenetic relation to closely related bacterial species also isolated from porcine nasal swab samples, Figure S10: Position of isolates 6a2I-BL10, 1a6R-MA01, 1a2R-BL09b, and 6a6R-CH12, in phylogenetic relation to closely related bacterial species also isolated from porcine nasal swab samples, Figure S11: Position of isolates 4a3I-BL07, 1a4I-BL06a, 4a1I-CH08, 7a2I-BL11, 7a1R-anCH19, and 1a6R-CH08an in phylogenetic relation to closely related bacterial species also isolated from porcine nasal swab samples, Table S1: Phenotypic antimicrobial susceptibility profiles of *E. coli* isolates from farms #1 to #3, Table S2: Phenotypic antimicrobial susceptibility profiles of *E. coli* isolates from farms #4 to #7.
