# Supplementary material for: The Porcine Nasal Microbiota with Particular Attention to Livestock-Associated Methicillin-Resistant Staphylococcus aureus in Germany—A Culturomic Approach
Source: Microorganisms. 2020 Apr 4;8(4):514. doi: 10.3390/microorganisms8040514 (PMC7232296; doi:10.3390/microorganisms8040514)
Supplement: Supplementary file 1 [file microorganisms-08-00514-s001.zip › Supplementary Tables.pdf]

microorganisms

MDPI

Table 1. Phenotypic antimicrobial susceptibility profiles of *E. coli* isolates from farms #1 to #3.

Farm	Individual	Habitat	Resistance Profile[a]	# of Isolates
#1	#1	Nasal cavity	AMP, AMC, AMO, PIP, AMS, TRS	4
		Snout surface	AMP, AMC, AMO, PIP, AMS, TRS	2
			-	1
	#5	Nasal cavity	TET	2
		Snout surface	AMP, AMC, AMO, PIP, AMS, TRS, TET	2
	#6	Nasal cavity	AMP, AMC, AMO, PIP, AMS, TRS, TET	2
			TET	1
#2	#1	Nasal cavity	AMP, AMC, AMO, PIP, AMS, TRS, TET	4
	#2	Nasal cavity	TRS	1
#3	#1	Snout surface	-	1
	#2	Nasal cavity	AMP, AMC, AMO, CIP, LEV, MOX, PIP, AMS, TRS, TET	2
			AMP, AMC, AMO, CIP, LEV, MOX, PIP, AMS	6
			AMP, AMO, CIP, LEV, MOX, PIP, TRS, TET	1
		Snout surface	AMP, AMC, AMO, CIP, LEV, MOX, PIP, AMS, TRS, TET	3
			AMP, AMC, AMO, CIP, LEV, MOX, PIP, AMS, PIT	1
			AMP, AMC, AMO, CIP, LEV, MOX, PIP, AMS	3
	#3	Nasal cavity	AMP, AMC, AMO, CIP, LEV, MOX, PIP, AMS, TET	1
		Snout surface	AMP, AMO, PIP, TET	1
			TRS, TET	1
			MOX	4
			-	1

[a] as determined by Vitek 2 using cards AST-N214; AMP, ampicillin; AMC, amoxicillin-clavulanate; AMO, amoxicillin; AMS, ampicillin-sulbactam; CIP, ciprofloxacin; CZO, cefazolin; GEN, gentamicin; LEV, levofloxacin; MOX, moxifloxacin; PIP, piperacillin; PIT, piperacillin-tazobactam; TET, tetracycline; TRS, trimethoprim/sulfamethoxazol

Table 2. Phenotypic antimicrobial susceptibility profiles of *E. coli* isolates from farms #4 to #7.

Farm	Individual	Habitat	Resistance Profile[a]	# of Isolates
#4	#1	Nasal cavity	TET	1
			-	1
		Snout surface	-	3
	#3	Nasal cavity	AMP, AMC, AMO, PIP, AMS, TET	1
			TET	2
			-	1
		Snout surface	-	1
#5	#1	Snout surface	AMP, AMC, AMO, GEN, PIP, AMS, TRS, TET, PIT	1
			AMP, AMC, AMO, PIP, AMS, TRS, TET, PIT	1
			AMP, AMC, AMO, PIP, AMS, TRS, TET	1
	#2	Snout surface	AMP, AMC, AMO, CIP, LEV, MOX, PIP, AMS, TRS, TET, PIT	1
			AMP, AMC, AMO, GEN, PIP, AMS	1
	#3	Nasal cavity	AMP, AMC, AMO, PIP, AMS, TRS	6
		Snout surface	AMP, AMC, AMO, PIP, AMS, TRS, TET	2
			AMP, AMC, AMO, PIP, AMS, TRS	2
#6	#1	Nasal cavity	TRS, TET	7
		Snout surface	TRS, TET	5
	#2	Nasal cavity	AMP, AMC, AMO, CZO, PIP	1
			-	1
		Snout surface	TRS, TET	1
			-	2
	#3	Nasal cavity	TRS, TET	3
#7	#1	Snout surface	-	5
	#2	Nasal cavity	-	1

[a] as determined by Vitek 2 using cards AST-N214; AMP, ampicillin; AMC, amoxicillin-clavulanate; AMO, amoxicillin; AMS, ampicillin-sulbactam; CIP, ciprofloxacin; CZO, cefazolin; GEN, gentamicin; LEV, levofloxacin; MOX, moxifloxacin; PIP, piperacillin; PIT, piperacillin-tazobactam; TET, tetracycline; TRS, trimethoprim/sulfamethoxazol
