# Supplementary figures and images for: The Porcine Nasal Microbiota with Particular Attention to Livestock-Associated Methicillin-Resistant Staphylococcus aureus in Germany—A Culturomic Approach
Source: Microorganisms. 2020 Apr 4;8(4):514. doi: 10.3390/microorganisms8040514 (PMC7232296; doi:10.3390/microorganisms8040514)

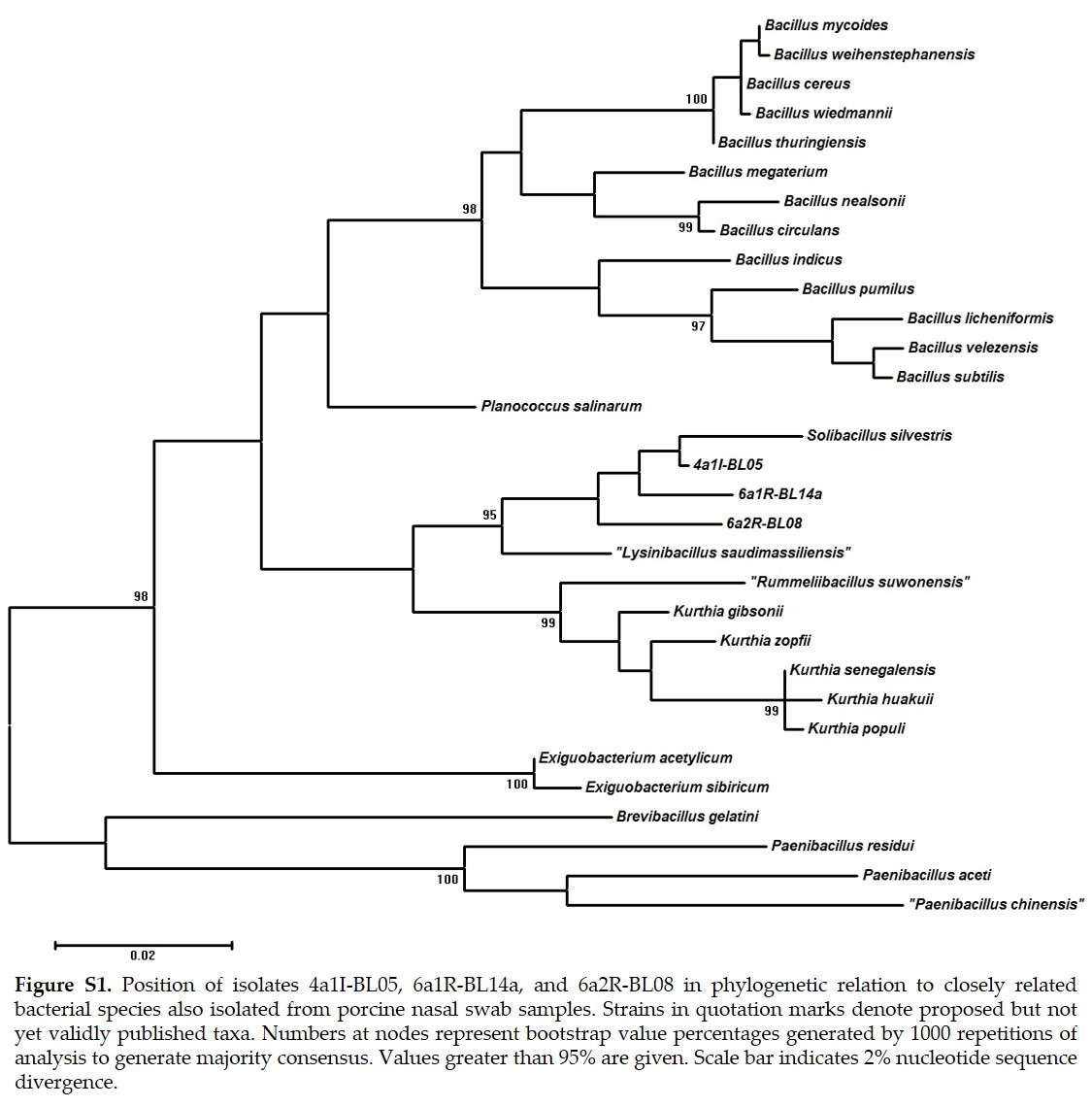

Supplement: Supplementary file 1 [file microorganisms-08-00514-s001.zip › Figure S1.jpg]

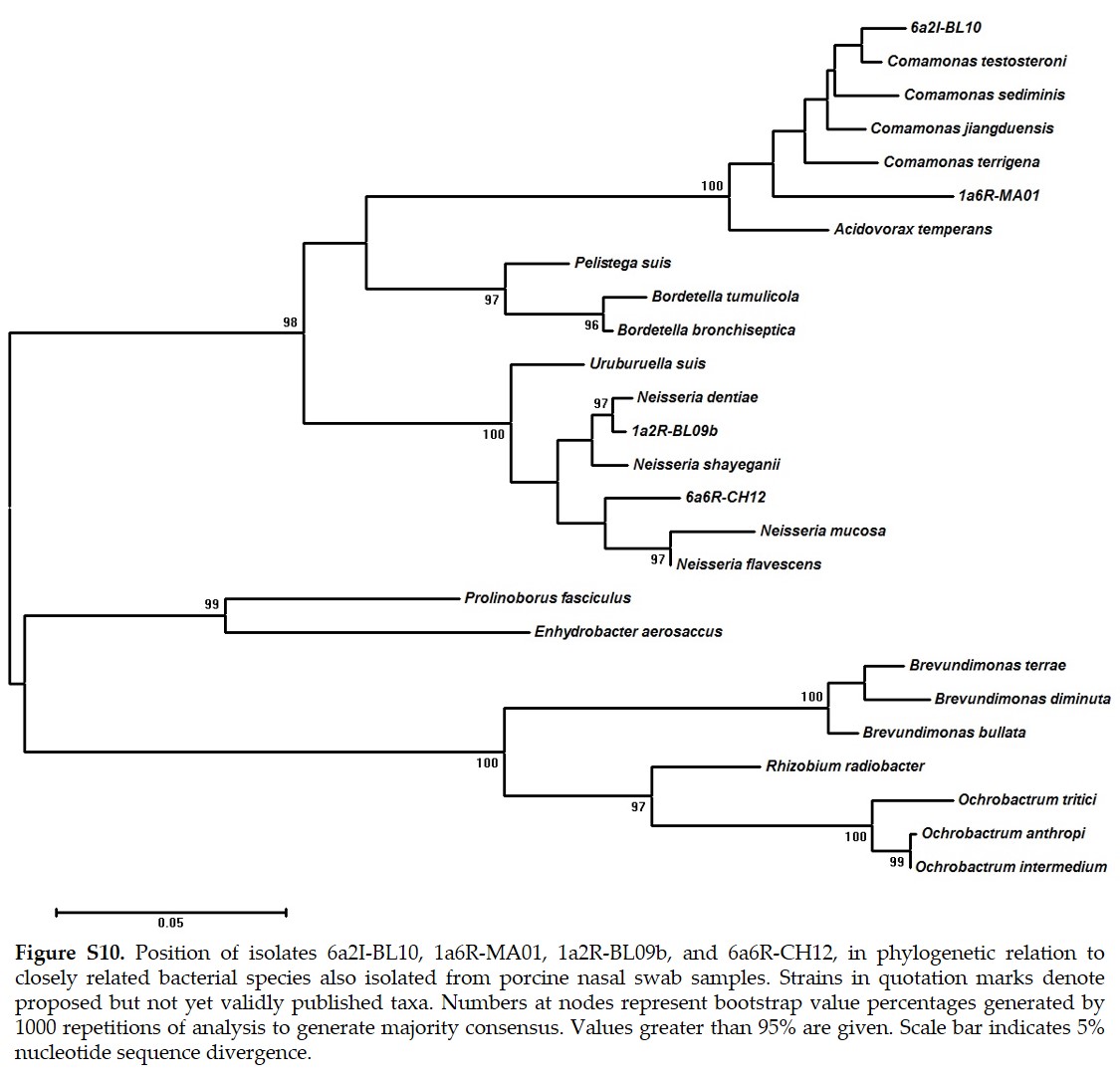

Supplement: Supplementary file 1 [file microorganisms-08-00514-s001.zip › Figure S10.jpg]

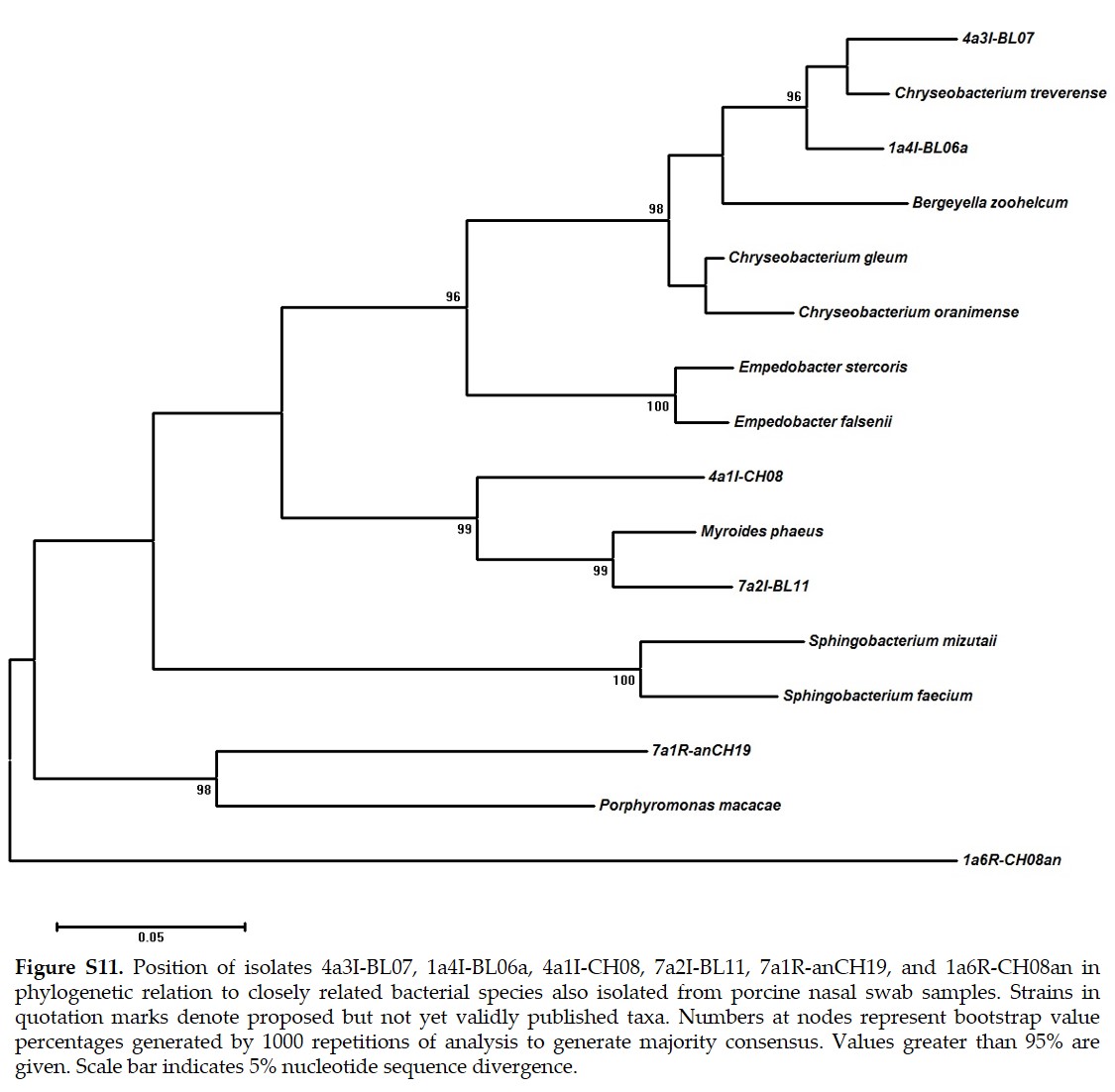

Supplement: Supplementary file 1 [file microorganisms-08-00514-s001.zip › Figure S11.jpg]

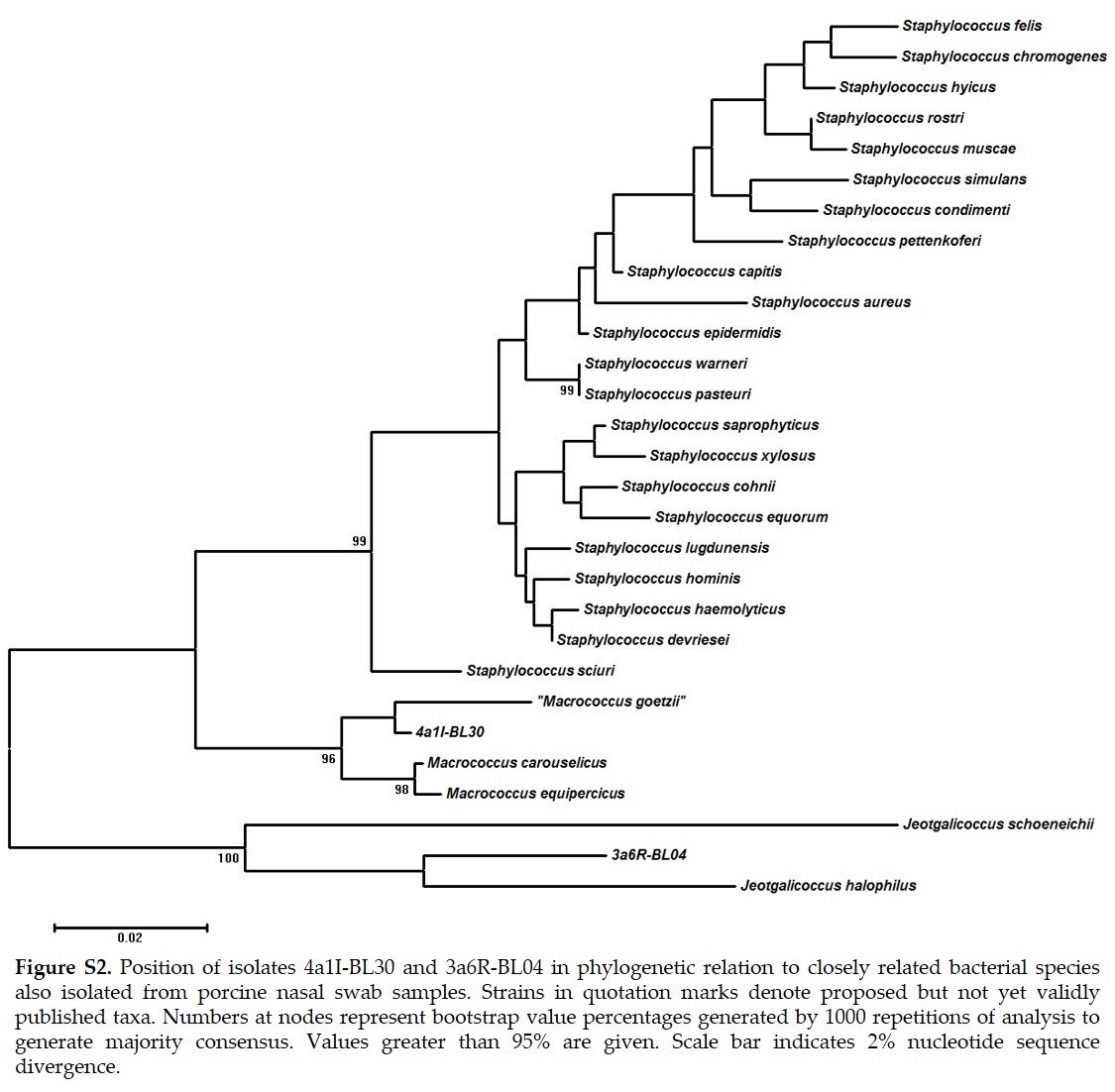

Supplement: Supplementary file 1 [file microorganisms-08-00514-s001.zip › Figure S2.jpg]

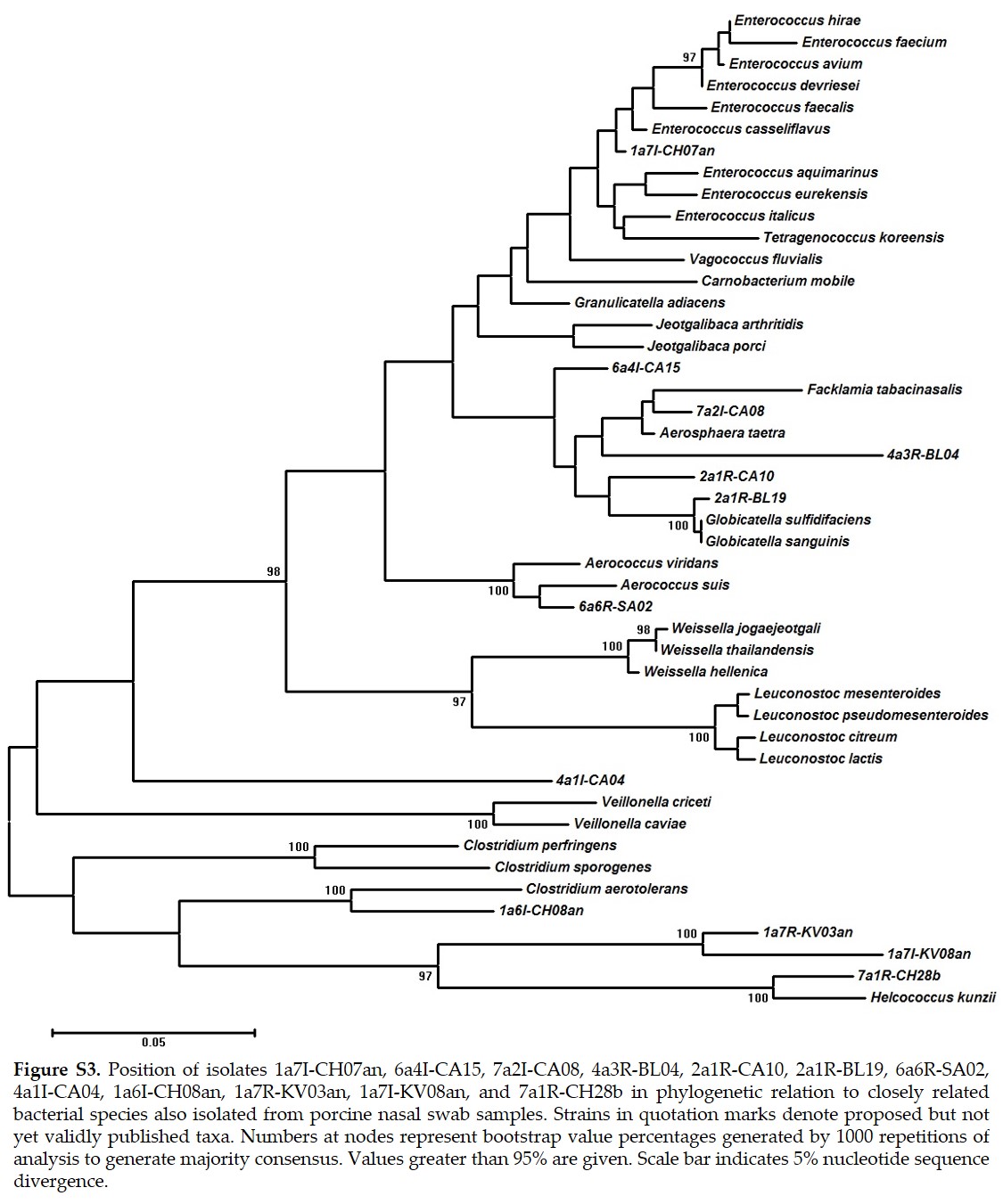

Supplement: Supplementary file 1 [file microorganisms-08-00514-s001.zip › Figure S3.jpg]

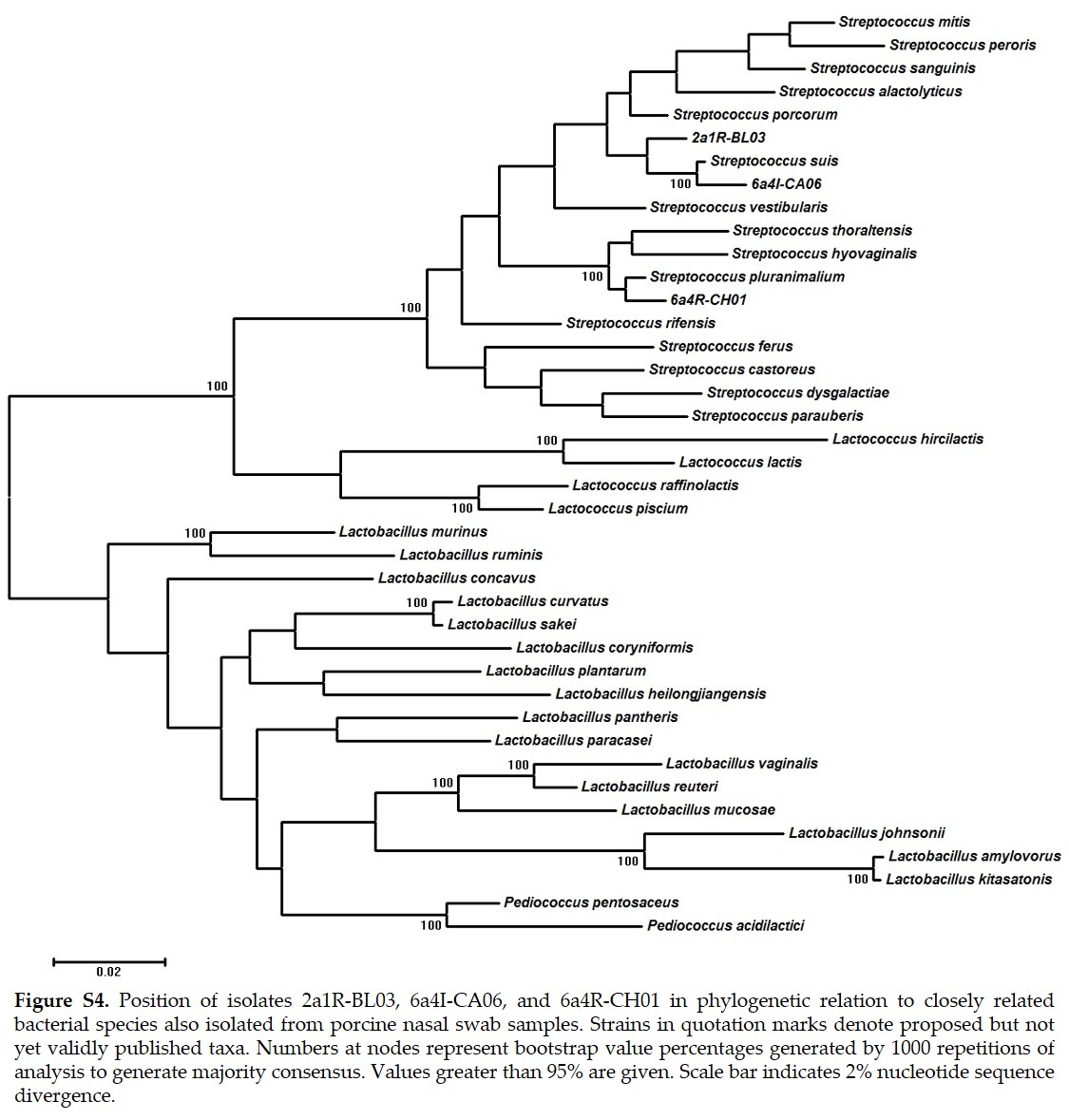

Supplement: Supplementary file 1 [file microorganisms-08-00514-s001.zip › Figure S4.jpg]

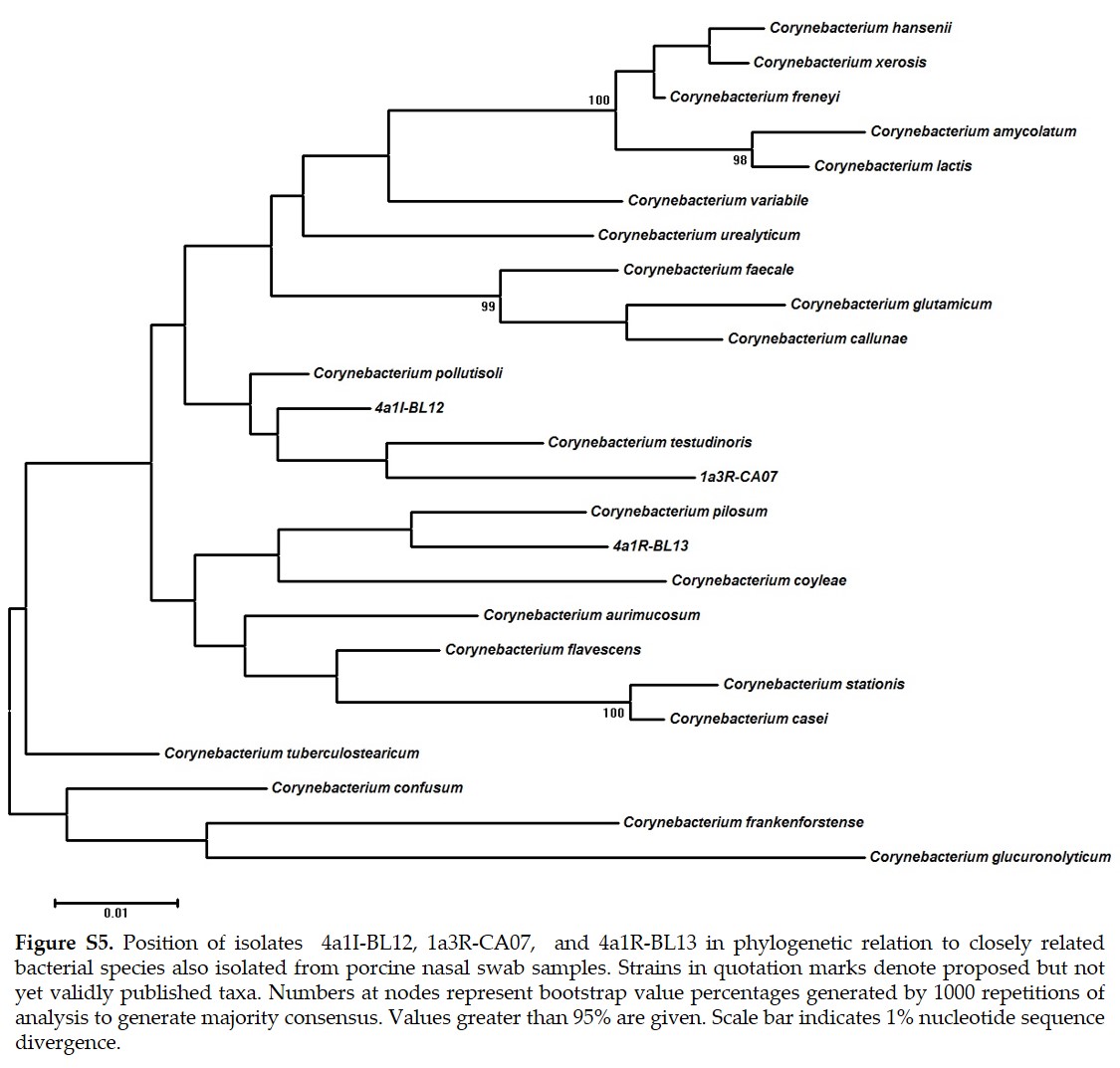

Supplement: Supplementary file 1 [file microorganisms-08-00514-s001.zip › Figure S5.jpg]

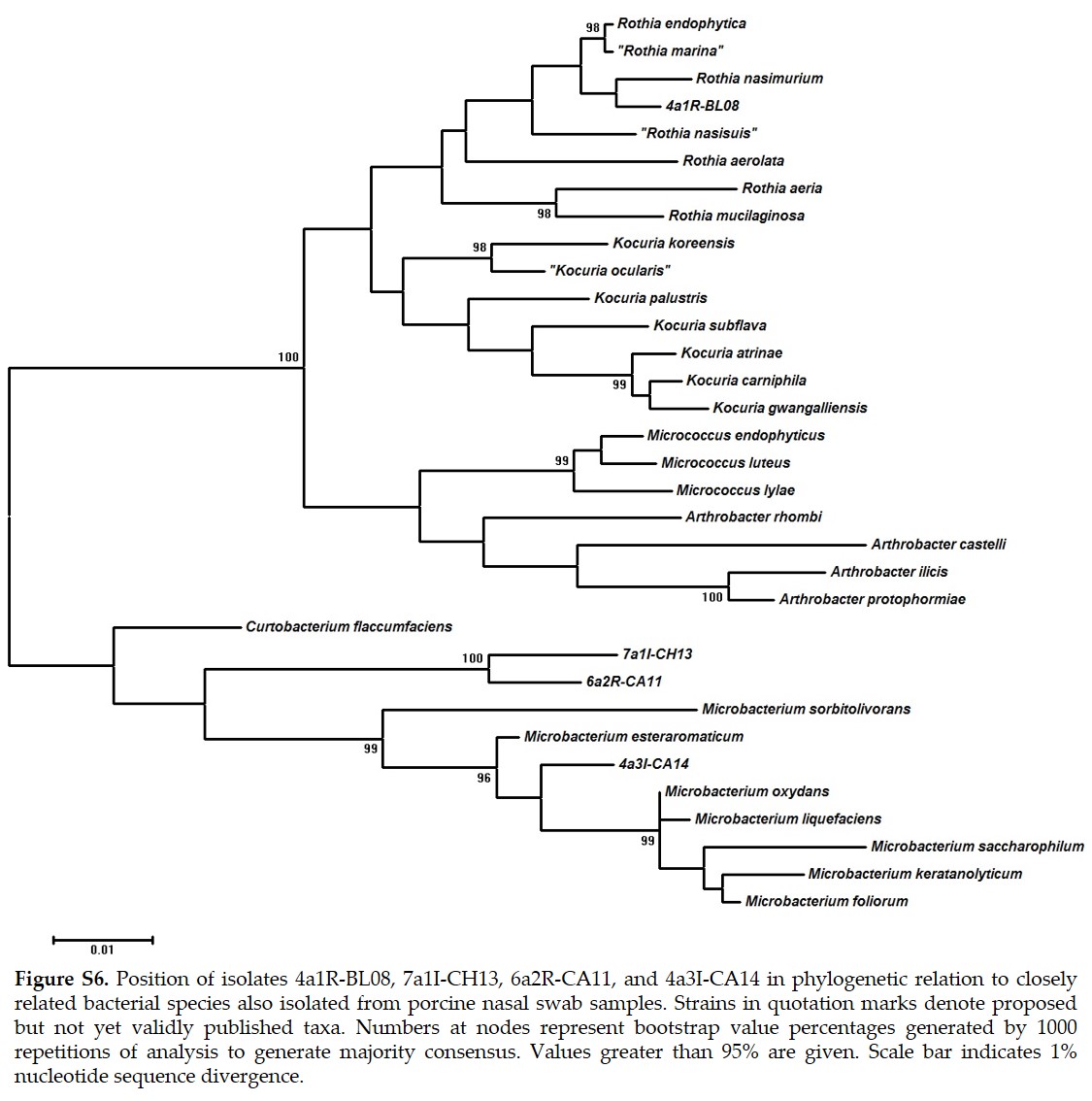

Supplement: Supplementary file 1 [file microorganisms-08-00514-s001.zip › Figure S6.jpg]

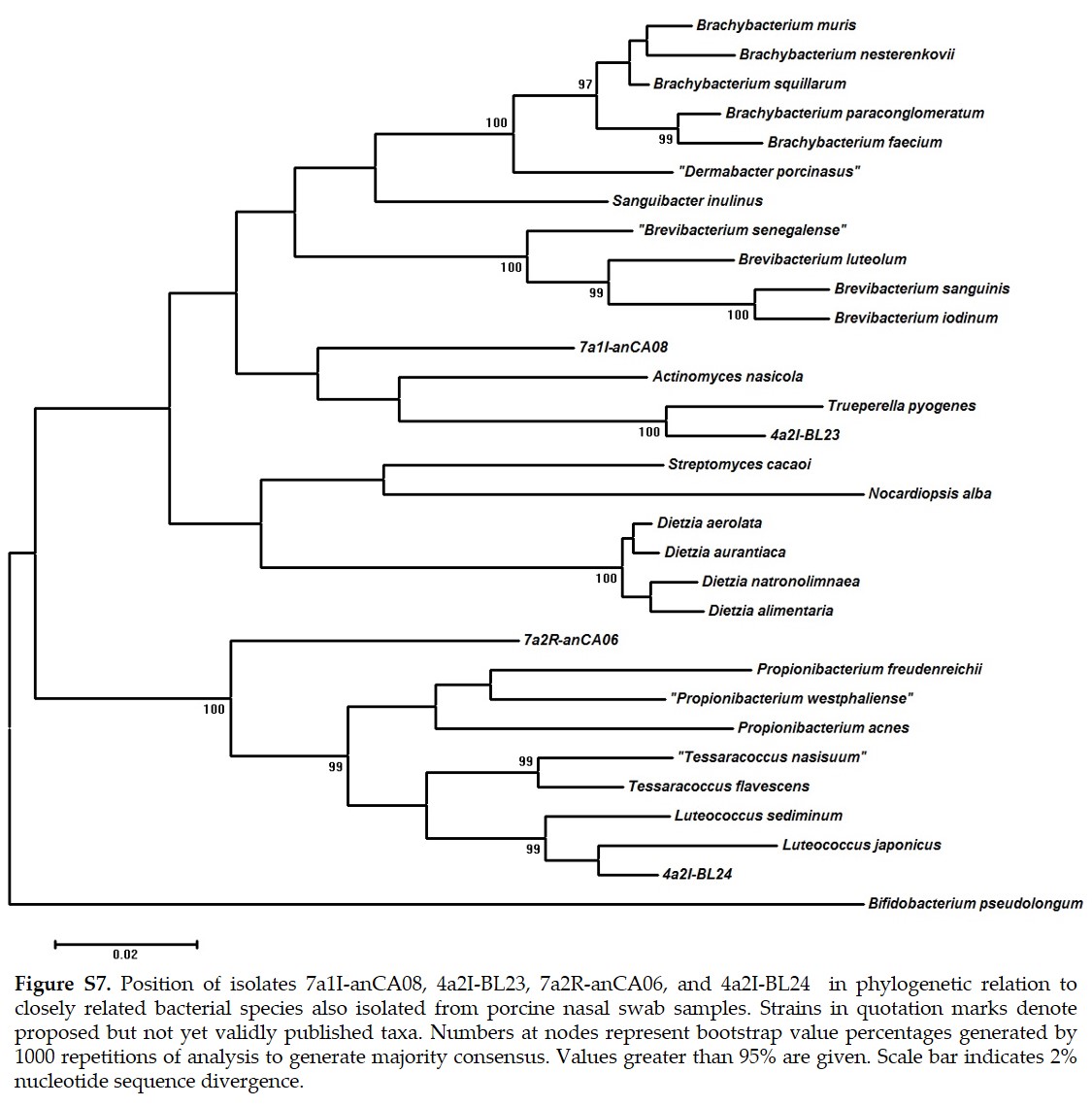

Supplement: Supplementary file 1 [file microorganisms-08-00514-s001.zip › Figure S7.jpg]

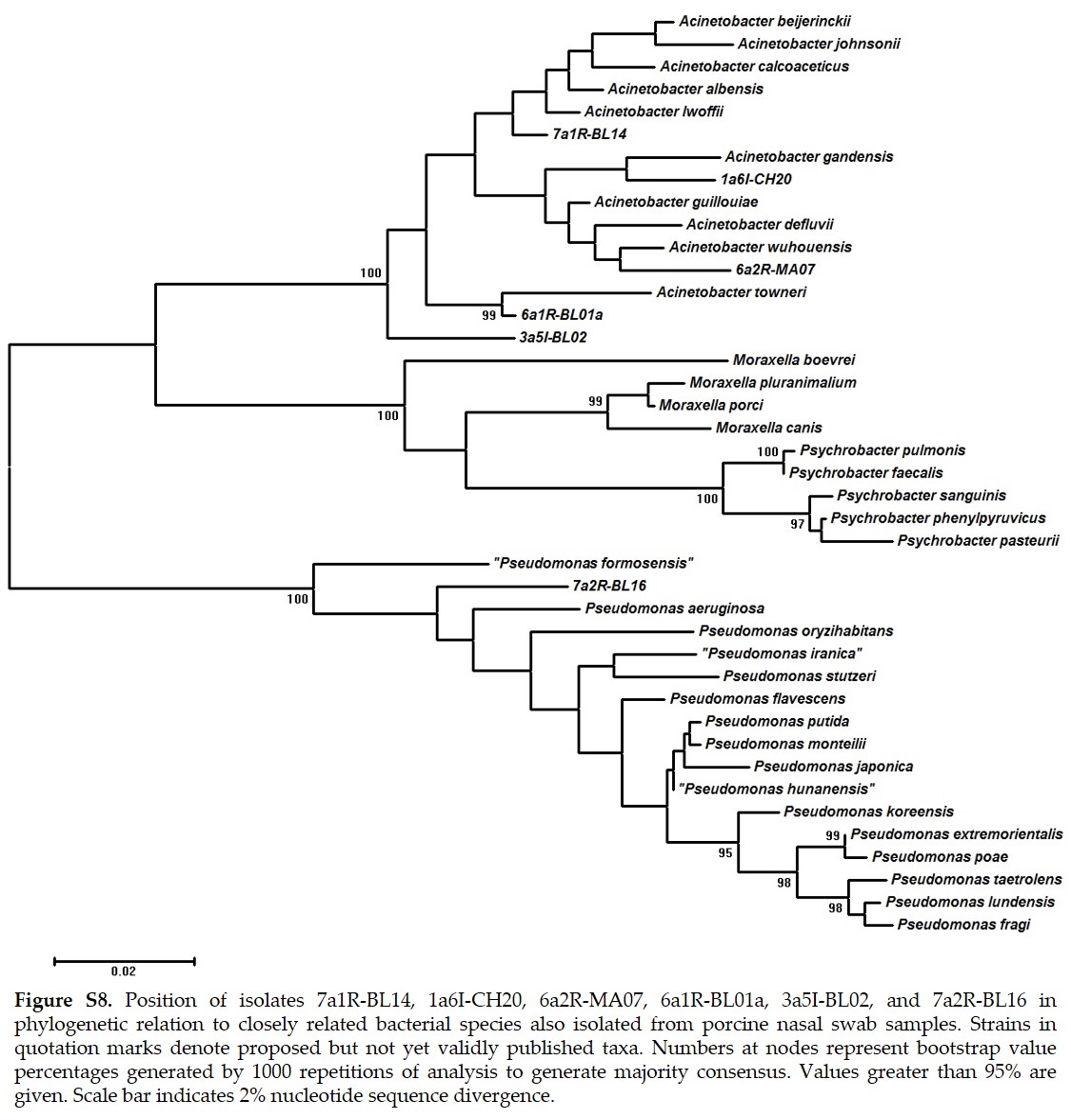

Supplement: Supplementary file 1 [file microorganisms-08-00514-s001.zip › Figure S8.jpg]

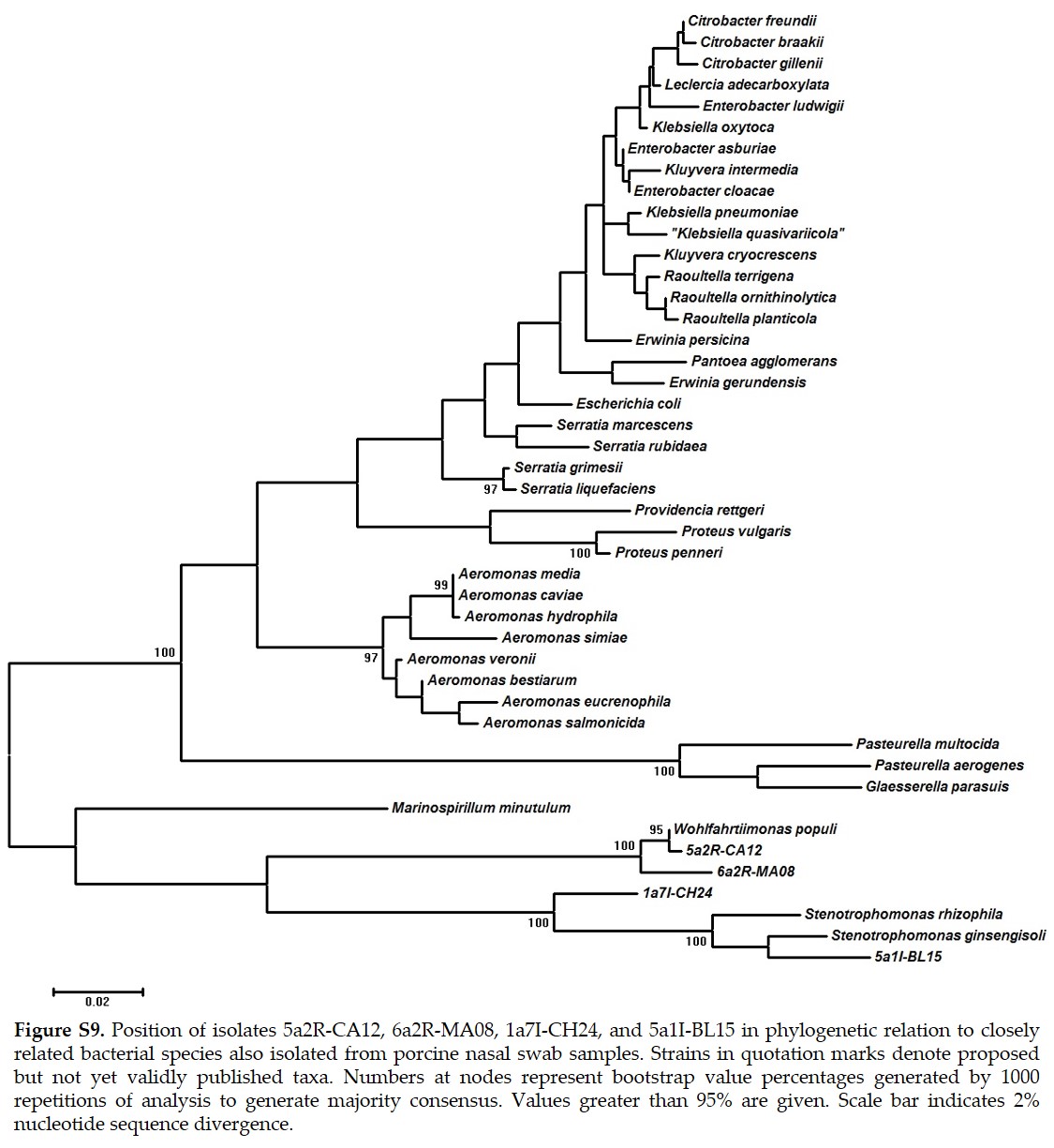

Supplement: Supplementary file 1 [file microorganisms-08-00514-s001.zip › Figure S9.jpg]
